# Methylenedioxy Piperamide-Derived Compound D5 Regulates Inflammatory Cytokine Secretion in a Culture of Human Glial Cells

**DOI:** 10.3390/molecules27113527

**Published:** 2022-05-30

**Authors:** Sajad Shahbazi, Tara Zakerali

**Affiliations:** 1 BRAINCITY, Neurobiology Lab, Nencki Institute of Experimental Biology, 02-093 Warszawa, Poland; 2 Nencki Institute of Experimental Biology, 02-093 Warszawa, Poland; tarazakerali@gmail.com

**Keywords:** neuroinflammation, neurodegenerative diseases, NF-κB, pro-inflammatory cytokines, piperine, piperamide

## Abstract

Neuroinflammation is the cornerstone of most neuronal disorders, particularly neurodegenerative diseases. During the inflammatory process, various pro-inflammatory cytokines, chemokines, and enzymes—such as interleukin 1-β (IL1-β), tumor necrosis factor-α (TNF-α), interleukin 6 (IL-6), inducible nitric oxide synthases (iNOS), inhibitory kappa kinase (IKK), and inducible nitric oxide (NO)—are over-expressed in response to every stimulus. **Methods**: In the present study, we focused on the anti-neuroinflammatory efficacy of (2E,4E)-N,5-bis(benzo[d][1,3]dioxol-5-yl)penta-2,4-dienamide, encoded D5. We investigated the efficacy of D5 on the upstream and downstream products of inflammatory pathways in CHME3 and SVG cell lines corresponding to human microglia and astrocytes, respectively, using various in silico, in vitro, and in situ techniques. **Results**: The results showed that D5 significantly reduced the level of pro-inflammatory cytokines by up-regulating PPAR-γ expression and suppressing IKK-β, iNOS, NO production, and NF-κB activation in inflamed astrocytes (SVG) and microglia (CHME3) after 24 h of incubation. The data demonstrated remarkably higher efficacy of D5 compared to ASA (Aspirin) in reducing NF-κB-dependent neuroinflammation. **Conclusions**: We observed that the functional-group alteration had an extreme influence on the levels of druggability and the immunomodulatory properties of two analogs of piperamide, D5, and D4 ((2E,4E)-5-(benzo[d][1,3]dioxol-5-yl)-N-(4-(hydroxymethyl)phenyl)penta-2,4-dienamide)). The present study suggested D5 as a potential anti-neuroinflammatory agent for further in vitro, in vivo, and clinical investigations.

## 1. Highlights

- D5 reduced the level of pro-inflammatory cytokines, through up-regulating PPAR-γ expression, suppressing IKK-β, iNOS, NO production, and NF-κB activation in inflamed astrocytes (SVG) and microglia (CHME3) after 24 h of incubation.
- D5 showed higher efficacy than ASA on the inhibition of the NF-κB pathway leading to neuroinflammation.
- D5 acts as a potent anti-neuroinflammatory agent.

## 2. Introduction

Inflammation in the central nervous system (CNS) is the main cause of various neurodegenerative disorders [1]. Neuroinflammation is mediated by glial cells. It is reported that IKK-β, NO, and TNF-α signal molecules are necessary for activating glial cells through the NF-κB signaling pathway in inflammatory progression. Following the glial cells’ over-activation, the cascade of pro-inflammatory cytokines, such as IL-1β, IL-6, TNF-α, and extreme production of NO, may contribute to the neuronal disorder, death, and tissue damage in the brain [1]. The mentioned issues are supported by several in vivo and in vitro reports indicating that the LPS stimulation of glial cells resulted in neuronal toxicity mediated by neuroinflammation [2]. Therefore, the neuroinflammation mediated by the pathological stimulation of glial cells is considered a potential therapeutic target for many neurological disorders [3]. The neuroinflammatory process, due to its critical role in most pathophysiological disorders in the brain, is a crucial part of most neurological disorder research. Thus, a deep understanding of the inflammatory pathway and its role in a specific disorder may contribute to early-bird brain disorder treatments. One of the promising treatments for most neurological disorders caused by neuroinflammation is controlling the inflammatory pathways in the initial steps [4]. Anti-inflammatory medicines such as non-steroidal anti-inflammatory drugs (NSAIDs) alleviate or cease the neuroinflammatory process, and reduce or delay the progression of neurodegenerative disorders, e.g., Alzheimer’s disease [5]. However, due to the high side-effect incidence of NSAIDs, there is a need to find similar or better effective drugs with lower side-effect incidence [5].

Recently, natural products (NPs) have attracted the attention of many pharmacists and pharmacognosists for treating inflammation-related disorders in the central nervous system. Piperine, as a well-known NP, is an alkaloid extracted from black pepper (*Pipernigrum*) [6]. Piperine’s chemical structure consists of a 2,3-dihydro-1H-indene bond to piperidine via a (3E,5E)-hepta-3,5-dien-2-one linker to the nitrogen atom of piperidine [7]. The main characteristic of piperine is high absorption and bioavailability through the gastrointestinal system in the human body [8]. Additionally, its great immunomodulatory function makes it an ideal compound for drug development purposes [7]. 

Piperine demonstrated significant anti-inflammatory efficacy through various in vitro and in vivo studies [9,10,11]. There are various reports regarding the impact of piperine and its amide derivatives on the suppression of the inflammatory response by immune cells [12]. However, the biological applications of piperine are limited due to its acute toxic effect and poor solubility in aqueous environments [13]. It is reported that piperoyl-amino conjugates have better biological activity than piperine [14]. In addition, pharmacokinetic studies demonstrated that piperine metabolism in humans results in retaining a methylenedioxy ring and conjugated double bonds while the piperidine ring in the piperine structure forms a propionic acid group [15]. The metabolism of piperine produces metabolites with better solubility and less toxicity [16]. 

Therefore, in the present study, we modified the piperine structure using a methylenedioxy ring as a replacement for piperidine residue and developed a novel piperamide derivative called (2E,4E)-N,5-bis(benzo[d][1,3]dioxol-5-yl)penta-2,4-dienamide (D5). Then, we investigated the role of D5 on the LPS inducing inflammatory responses in the glial cell lines. We compared the physicochemical and toxicological properties of D5 with those of our previously developed piperamide, (2E,4E)-5-(benzo[d][1,3]dioxol-5-yl)-N-(4-(hydroxymethyl)phenyl)penta-2,4-dienamide) called D4, to determine the impact of functional-group alteration on the pharmacological properties of piperamides. In addition, we tested the efficacies of D5 and D4 along with a commonly used non-selective, non-steroidal, anti-inflammatory drug (Aspirin, encoded ASA); we performed this test to suppress the neuroinflammatory pathways throughout the various molecular assays on the two types of neuroglial cell lines, including immortalized human microglia (CHME3) and astrocyte (SVG) cell lines. Finally, the efficacies of D5 and D4 were compared to investigate the anti-neuroinflammatory profile change and the structural improvement of the novel methylenedioxy piperamide for its drug-likeness properties. 

## 3. Materials and Methods

### 3.1. In Vitro Study

#### 3.1.1. Cell Culture 

SV40 (human immortalized astrocyte (SVG)) and SV40 (human fetal brain-immortalized microglia) CHME3 cell lines (HMC3/ATCC) were sourced by Dr. Anirban Basu from the National Brain Research Centre (NBRC), Manesar, Haryana, India [17,18]. Both cell lines were plated in the 6-well plates with a cell density of 0.5 × 10^6^ cells/well. The medium used for culturing the cells was Dulbecco’s Modified Eagle Medium (DMEM: high glucose with glutamax/glutamine, Thermo Fisher, Odisha, India) supplemented with 10% heat-inactivated Fetal Bovine Serum (FBS), penicillin (100 U/mL), and streptomycin (100 μg/mL). After plating, cells were incubated at 37 °C in a humidified atmosphere of 5% CO_2_.

#### 3.1.2. Cell Treatment

The stock solutions of tested compounds were prepared by dissolving 1 mg of each compound, including D5 and ASA (acetylsalicylic acid, Aspirin^®^, Odisha, India), in 1 mL of DMSO (Dimethyl Sulfoxide, Thermo Fisher, Odisha, India) (1 µg/µL) and sterilized with a 0.2 µm filter paper (Thermo Fisher, Odisha, India). For the treatments, the desired concentration of each compound was prepared through serial dilution of each stock solution to obtain 0.71 µM of D5 and 52.73 µM of ASA. To reduce the adverse effect of DMSO on our experiment result, we used the minimum concentration, using serial dilution with a max dilution of 1:4000 of DMEM medium in the treated wells. The selected doses of the compounds were counted from the homology study using the practical EC50 value of the known drug molecules obtained from the FDA database.

Cells were seeded for 48 to 72 h with a medium replacement before the treatment. Both cell lines were in their 3rd passages. Before experiments, the cell density of both cell lines, CHME3 and SVG, with doubling times of 48 h and 72 h, respectively, was around 1 × 10^6^ cells/well. The next day, cells were pre-treated with D5 and ASA compounds for 2 h before being subjected to LPS (100 ng/mL, Sigma-Aldrich, Odisha, India) for 24 h [19]. The D5- and ASA-treated cells, as the trial groups, were compared to the LPS-stimulated cells as a positive control, and normal untreated cells as a negative control. 

#### 3.1.3. Cytoprotective Effect of D5 in the Selected Concentration

An MTT (3-[4,5-dimethylthiazol-2-yl]-2,5 diphenyl tetrazolium bromide) assay (Sigma-Aldrich, Odisha, India) was employed to evaluate the cytoprotection efficacy of D5 for the glial cells against inflammation leading to cell damage and apoptosis. The procedure was performed according to the manufacturer’s standard protocol, with slight modification [20]. Briefly, cells of both cell lines were plated on the 24-well plates (1 × 10^5^ cells/well) and incubated for 24 h. Cells were treated with LPS, along with D5 and ASA in the selected doses, for 24 h, in 3 replications, and wells were kept for positive and negative controls. Then, 250 µL of MTT at a concentration of 2 mg/mL was added to each well. After 2 h of incubation, the mixture was decanted, and 500 µL of DMSO was added to dissolve the formazan reagent and shacked for 30 min at room temperature (RT). Finally, a measurement of absorbance (OD) was performed at 540 nm using a Mark™ Microplate Absorbance Spectrophotometer (Bio-Rad, Odisha, India). 

#### 3.1.4. NO Quantification 

Glial cells secrete nitric oxide during inflammatory events, and the level of NO_2_ is considered an indicator of the level of inflammatory response by the glial cells. For the estimation of the level of NO generation, a Griess reagent (Sigma-Aldrich, Odisha, India) was used based on the previously reported protocol, with slight modification [21]. In this method, a diazotizing reagent such as sulfanil amide (SA) is used to treat nitrite in acidic media to form a transient diazonium salt. Then, the diazonium salt interacts with a coupling reagent, N-naphthyl-ethylenediamine (NED), to form a stable azo compound. The generated intense purple color of the product can be measured for quantification of the nitrite concentration. Finally, the density of generated colors can be read out at 540 nm.

Briefly, the cells, at a concentration of 1 × 10^5^ cells/well, were plated on 24-well plates containing 500 µL of DMEM in each well for 24 h. Next, the cells were treated with LPS (100 ng/mL) in the presence or absence of D5 and ASA for an extra 24 h. Subsequently, an equal amount of Griess reagent was added to each well and mixed well with the supernatants. After 10 min of incubation at RT, the measurement of absorbance was performed using the xMark™ Microplate Absorbance Spectrophotometer (Bio-Rad, Odisha, India) at 540 nm.

#### 3.1.5. Isolation of Total Proteins

The total protein was extracted from cells according to the standard protocol [22] after 24 h of incubation in the presence of drugs. The experiment was performed with4 replicates per experimental group and 3 repeats per replicate. Briefly, the lysates of adhered cells were prepared by adding 0.5 mL of RIPA lysis buffer (Sigma-Aldrich, Odisha, India) (for up to 5 × 10^6^ cells) to pre-washed cells with cold PBS in the cultured dishes. After homogenizing by pipetting 20 times, lysates were transferred into a 1.5 mL Eppendorf tube. Following incubation for 5 min at 4 °C, lysates were centrifuged at 14,000 rpm for 15 min at 4 °C. The supernatant was collected and the protein concentration was estimated using the Lowry assay (Total Protein Kit Micro-Lowry, Sigma-Aldrich, Odisha, India) according to the manufacturer protocol, with minor modification.

#### 3.1.6. Western Blot Analysis

The obtained proteins were denatured by heating them at 100 °C for 5 min, and around 20 to 40 µg of the aliquots were loaded into the 7% polyacrylamide SDS-PAGE gels (sodium dodecyl sulfate-polyacrylamide gel electrophoresis), along with a molecular weight marker. After transferring proteins to a PVDF membrane (Immobilon®-P PVDF Membrane Millipore, Sigma-Aldrich, Odisha, India), the PVDF strips were washed with Tris-buffered saline (TBS: 20 mM Tris, 0.15 M NaCl, pH 7.5) for 5 min, followed by 10 s methanol soaking and air drying. The dried membrane strips were incubated overnight at 4 °C with primary antibodies, including rabbit anti-Phospho-IκB alpha (S32) (1:4000, Imgenex, Bhubaneswar, Odisha, India), rabbit anti-inducible nitric oxide synthase (iNOS) (4.66 µg/5 mL, Invitrogen, Thermo Fisher, Odisha, India) and rabbit anti-Glyceraldehyde 3-phosphate dehydrogenase (GAPDH, 5 µg/5 mL, Invitrogen, Thermo Fisher, Odisha, India) antibodies in 5% skim milk/well. After washing twice by 0.1% TBST for 15 s, the strips were incubated for one hour with horseradish peroxidase (HRP)-labeled secondary goat anti-mouse antibody (1:5000, Thermo Fisher, Odisha, India) at RT. Then, strips were washed and covered using ECL (Enhanced luminol-based chemiluminescent substrate, Sigma-Aldrich, Odisha, India), in a ratio of 1:1 (2 mL for each block). After 5 min, they were exposed to the Kodak X-ray film (Sigma-Aldrich, Odisha, India) for 15 s to 5 min, and the density of bands was quantified usingthe Fiji module of ImageJ wizard [23]. The analyzed data were based on the ratio between the band density of the interested protein and the loading control protein (GAPDH).

#### 3.1.7. Enzyme-Linked Immunosorbent Assay (ELISA)

In the present study, we used an ELISA assay for two purposes: (**a**) In the pre-screening stage, the optimum drug dosage was determined using a IL-6 Human ELISA Kit, High Sensitivity (Invitrogen, Thermo Fisher, Odisha, India, cat: **BMS213HS**), and an IFN gamma Human ELISA Kit (Invitrogen, Thermo Fisher, Odisha, India, cat: **BMS228**); and (**b**) in the screening stage, the assay was employed for drug efficacy on cytokine expression using a TNF-α, and IL-1β Human ELISA Kit, Invitrogen, Thermo Fisher, Odisha, India (cats: **BMS223HS** and **KHC0011**, respectively). The collected medium from each well of cell culture plates was screened for the level of secreted pro-inflammatory cytokines, including IL-6, IFN-γ, TNF-α, and IL-1β, using sandwich ELISA kits for human samples according to the manufacturer standard protocol, with minor modifications (Invitrogen, Thermo Fisher, Odisha, India) [24]. Briefly, around 50 µL of each biotinylated anti-human IL-6, IFN-γ, TNF-α, and IL-1β antibody, along with an equal amount of each sample and respective standard, were added to a 96-well ELISA plate and incubated for 90 min. Following washing 4 times with 1min intervals, the secondary HRP-conjugated antibody was added to each sample and incubated for 30 min. Then, the samples were washed and the chromogen solution was added. After 30 min of incubation in a dark condition, the stop solution was added and a measurement of absorbance was performed using the xMark™ Microplate Absorbance Spectrophotometer (Bio-Rad, Odisha, India) at 450 nm.

#### 3.1.8. Immunocytochemistry

Cells were cultured on gelatin-coated (Sigma-Aldrich, Odisha, India) coverslips and treated with D5 and ASA, 2 h before the LPS stimulation. After 60 min, cells were fixed in 3% paraformaldehyde (Thermo Fisher, Odisha, India) for 20 min, and permeabilized for 1 h with 0.1% triton-X-100 (Sigma-Aldrich, Odisha, India). Blocking was carried out with 2.5% normal horse serum for 20 min. Incubation with primary antibodies was performed at 4 °C overnight in blocking solution before washing and detection with a secondary antibody. The primary antibodies used were 5 µg/mL mouse monoclonal NF-κB p65 antibody (Thermo-Fisher, Poland). The secondary antibodies used were 10 µg/mL goat anti-mouse IgG Alexa Fluor488 (Thermo Fisher, Odisha, India). Controls were performed using secondary antibodies alone. Coverslips were mounted using Vecta shield Hardest containing DAPI (4′,6-diamidino-2-phenylindole, Vector Laboratories, Odisha, India). Finally, the images were prepared using a Leica SP8 TCS confocal microscope with two laser channels, including green and blue (EX/EM: 488/550, and 359/457, respectively). The quantification of the nuclear translocation rate of activated NF-κB p65 was performed using Cellprofiler 4.2.1 software (Broad Institute of MIT and Harvard) [25]. 

Side by side, phospho-IκB-α tracking was performed after the fixation of cells. Then, all samples were stained non-specifically with 10% normal goat serum in PBS containing 1% BSA, for 20 min, at room temperature. Subsequently, samples were incubated overnight at 4 °C with monoclonal rabbit anti-Phospho-IκB alpha (S32) as the primary antibody (diluted by 1:4000, Imgenex, Bhubaneswar, Odisha, India). The next day, samples were incubated with Extr Avidin-HRP (1:500, Sigma-Aldrich, Odisha, India) for 1 h at room temperature. Finally, a DAB solution and Hematoxylin were added to develop the color. The microscopic images were obtained from an Olympus IX70 microscope (Olympus, Okoya, Japan) equipped with a digital camera (CC-12, Cell’ P Olympus Soft Imaging System GmbH, Hamburg, Germany).

#### 3.1.9. Total RNA Extraction and Quantitative Real-Time PCR (qPCR)

Total RNA was extracted from the test and control groups using HiPurA™ 96 Total RNA Purification Kit (Himedia, Odisha, India) based on the manufacturer protocol, with minor modifications [26]. The quality of the extracted RNA was confirmed by a TECAN Infinite M200 PRO spectrometer (Thermo Fisher, Odisha, India). Briefly, we dissolved 1 µg of total RNA in 13 µL of PCR-grade water containing 2 µL of Random Hexamer Primer (600 pmol/μL), provided by a Transcriptor First Strand cDNA Synthesis Kit (Roche, Life Science, Odisha, India), and heating for 10 min at 60 °C for denaturation. Then, we added an RT-mix containing 10 U of Transcriptor Reverse Transcriptase (Roche, Life Science, Odisha, India), 1 mM of each deoxyribonucleotide triphosphate (dNTPs, 10 mM each, Roche), 12 mM of random hexamer primer (600 pmol/mL, Roche, Life Science, Odisha, India), and 20 U Protector RNase inhibitor (40 U/mL, Roche, Life Science, Odisha, India) to the denatured RNA and incubated it for 10 min at 25 °C, followed by 30 min at 55 °C. Finally, we terminated the cDNA synthesis process by heating the mixture at 80 °C for 5 min and cooling it on ice [27]. Next, we added 1 µL of synthesized cDNA (100 ng) to a vial containing 0.5/0.5 µL of forward and reversed primers (300 nM each), 5 µL of 2X iTaq Universal SYBR Green Super mix (Bio-Rad, Odisha, India) [28], and 3 µL of DNase-free H_2_O (Bio-Rad, Odisha, India). We set the Step One RT-PCR machine (Applied Biosystems, Thermo Fisher, Odisha, India) for a total of 35 cycles under the following schemes: 30 s at 95 °C, 15 s at 95 °C, and 60 s at 60 °C. In the present study, the primers were designed and virtually tested for the self-complementary and PCR products using several online tools such as UCSC Genome Browser (developed by the University of California, Santa Cruz) [29], Pick Primer (NCBI) [30], Oligocalc (Oligonucleotide Properties Calculator, Northwestern University, Chicago, IL, USA) [31], and Primer3 (ELIXIR—European research infrastructure for biological information) [32]. All the primers were synthesized by Eurofins Company, including IKK-β (F: 5′-GTGCACAAGCAGACCAGTGT-3′, R: 5′-GCCCTTGGCAGTGTTGTAAT-3′), MIP (F: 5′-GCAACCAGTTCTCTGCATCA-3′, R: 5′-TTTCTGGACCCACTCCTCAC-3′), TNF-α (F: 5′-ACAAGGCTG CCCCGACTAC-3′, R: 5′-TGGAAGACTCCTCCCAGGTATATG-3′), PPAR-γ (F: 5′-GTTTGAGGGGGTAACAGCAA-3′, R: 5′-GCTAACTGCAGAGGGTGAGG-3′), and GADPH (F: 5′-ATGGGGGAAGGTGAAGGTCG-3′, R: 5′-GGGGTCATTGATGGCAACAATA-3′). The obtained results were computed using the Livak method [33].

### 3.2. The Pharmacological Properties of Methylenedioxy (D5) vs. Hydroxymethyl (D4) Derivatives of Piperamide 

In the present study, to investigate the effect of functional-group alteration of piperine’s chemical structure on its physicochemical, physiological and toxicological properties, we performed a series of side-by-side in silico and in vitro studies with the same experimental conditions using our previously and newly developed piperamides, hydroxymethyl (D4) and methylenedioxy (D5) derivatives, respectively. 

#### 3.2.1. In Silico Evaluation of Pharmacological Properties and Potential Inhibitory Nature

- Comparison of the physicochemical and toxicological properties

The ADEMT study was performed using the Qikprop tool of Schrodinger suite 2018 (Mannheim, Germany) [34] and TOPKAT approach in Accelrys Discovery studio 2.5 (Dassault Systemes BIOVIA, San Diego, CA, USA) [35] to evaluate the pharmacokinetics of both compounds. Various parameters were considered, such as **SASA**: the total solvent-accessible surface area (SASA) in square angstroms using a probe with a 1.4 Å radius; **FOSA**: the hydrophobic component of the SASA; **FISA**: The hydrophilic component of the SASA; **Volume**: The total solvent-accessible volume in cubic angstroms using a probe with a 1.4 Å radius; **donor HB**: the estimated number of hydrogen bonds that would be donated by the solute to water molecules in an aqueous solution; **accptHB**: the estimated number of hydrogen bonds that would be accepted by the solute from water molecules in an aqueous solution; **CNS**: the predicted central nervous system activity on a −2 (inactive) to +2 (active) scale; **QPlogPC16**: the predicted hexadecane/gas partition coefficient; **QPlogPoct‡**: the predicted octanol/gas partition coefficient; **QPlogPw**: the predicted water/gas partition coefficient; **QPlogPo/w**: the predicted octanol/water partition coefficient; **QPlogHERG**: the predicted IC50 value for blockage of HERG K+ channels; **QPlogBB**: the predicted brain/blood partition coefficient; **QPPMDCK**: the predicted apparent MDCK cell permeability in nm/sec; **QPlogKp**: the predicted skin permeability, log Kp; **QPlogKhsa**: the prediction of binding to human serum albumin; **HOA**: predicted qualitative human oral absorption: 1, 2, or 3 for low, medium, or high; **%HOA**: predicted human oral absorption on a scale of 0 to 100%; **ROF**: the number of violations of Lipinski’s rule of five; **ROT**: the number of violations of Jorgensen’s rule of three; **QPlogS**: the predicted aqueous solubility, log S; **QPPCaco**: the predicted apparent Caco-2 cell permeability in nm/sec; **#primary metabolites**: the number of likely metabolic reactions. **WOE**: the weight of evidence (carcinogenicity for humans), rodent carcinogenicity and mutagenicity; **LD50**: the lethal dose for rodents; **LC50**: the lethal concentration for rodents; **TD50**: the tolerated dose for rodents; and **EC50**: a concentration of a substance that causes adverse effects on 50% (EC50) of the test population of *Daphnia magna*, within a selected period, is used for predicting an optimal effective dosage of a druggable compound with a minimal cytotoxic effect.

b. Comparison of pharmacodynamic properties against IKK-β enzyme

IKK-β enzyme locates the upstream of almost all proinflammatory cytokine expression and acts as a key activator and modulator for NF-κB nuclear translocation and neuroinflammation [36]. Due to the critical role of the IKK-β enzyme in the phosphorylation of the inhibitory subunit of NF-κB (IκB-α), followed by activating the NF-κB complex in the cytosol and translocating into the nucleus [37], we selected the mentioned enzyme as a target protein for our in silico studies. 

The binding affinities of D4 and D5 for the active site of the IKK-β enzyme were evaluated using the GLIDE tool of Schrodinger suite 2018, to investigate the pharmacodynamic improvement due tothe functional-group modification of piperine. The level of docking scores and binding energies of both compounds for occupying the binding pocket of the IKK-β enzyme were compared, to determine the best functional-group modification for improving the efficacy and druggability of piperine.

Briefly, the IKK-β enzyme’s 3D structure (PDB ID: 4KIK) was downloaded from the RCSB Protein Data Bank (http://www.rcsb.org/pdb/) with X-ray diffraction resolutions of 2.83 Å [38,39]. The obtained PDB file was prepared with the Protein Preparation Wizard of Schrodinger suite 2018 using OPLS3e force-field (Schrödinger Suite; Epik version 4.6; Impact version 8.1; Prime module, Schrödinger, LLC, New York, NY, USA, 2018).

Next, the ligands were sketched using Chem BioDraw Ultra version 12.0 (Cambridge Soft suite 2010, USA) in a MOL-format file, and then transferred to Schrodinger suite 2018 for further in silico investigations. Then, all designed ligand structures were prepared with the LigPrep module of Schrodinger Suite 2018 using the OPLS3e force-field at a biologically relevant PH.

Finally, the Glide 8.1 module in the Extra Precision (XP) model and OPLS3e force-field were used for scoring the docking of ligands and the binding site of the IKK-β enzyme. The binding energy of each complex was measured using the Prime-MM-GBSA module (the molecular mechanics/generalized Born surface area) of Schrodinger Suite 2018 [40]. The 3D structure of each receptor–ligand complex was mapped via the XP visualizer approach of Schrodinger 2018, and the receptor surfaces were configured based on the electrostatic potential of residues in the binding pocket of protein by truncating the receptor surface at 5 Å from the ligand, with 20% transparency [41].

#### 3.2.2. In Vitro Druggability Evaluation of D5 vs. D4 on Neuroinflammatory Progress

Astrocytic glial (SVG) and microglial (CHME3) cells were treated with D4, D5, and ASA in their respective EC50 values (0.86 µM, 0.71 µM, and 52.73 µM, respectively) for 2 h, and then incubated with LPS (100 ng/mL) for 24 h. The efficacies of all compounds were compared using the pairwise Student’s *t*-test where *p* ≤ 0.05. The Student’s *t*-test was performed to compare the obtained results from various in vitro tests examining the efficacy of methylenedioxy piperamide and hydroxymethyl piperamide on the inflammatory pathways in glial cells of both CHME3 and SVG cell lines in the 24 h time frame. The level of significant differences between groups was determined to be *p* < 0.05. The in vitro tests consisted of assessing the level of cytoprotection using an MTT assay, NO quantification using a Griess reagent, protein secretion (iNOS and phospho-IκB) using Western blot analysis, cytokine secretion (IL-6, IFN-γ, TNF-α, and IL-1β) using an Enzyme-Linked Immunosorbent Assay (ELISA), and cytokine and enzyme gene expression (IKK-β, PPAR-γ, MIP, and TNF-α) using quantitative real-time PCR. 

### 3.3. Data Presentation and Statistical Analysis

A one-way ANOVA followed by post hoc analysis (Bonferroni Correction)using the Excel tool of Microsoft office 2007 (Microsoft, Redmond, WA, USA) [42], at a confidence level of 95% (α = 0.05), was used to compare groups, and the results were presented as the means ± S.D. A two-tailed *t*-test analysis was used to compare the efficacies of D5 and D4 at a confidence level of 95% (α = 0.05).

## 4. Results

### 4.1. In Vitro Pharmacological Property Evaluation of D5 against LPS-Induced Inflammation in the Glial Cells

D5 was tested for its druggability and anti-neuroinflammatory potency through various in vitro studies. The LPS pre-treated microglia and astrocytic glial cell lines, CHME3 and SVG, were used as in vitro PAMP (pathogen-associated molecular patterns) models of inflammogenesis in the human central nervous system. ASA, as a commonly used anti-inflammatory drug, was used for evaluating the level of drug potency of D5 as an anti-neuroinflammatory agent. The efficacy tests were performed according to the inhibitory potential of D5, compared to ASA, on the levels of the cytokines and chemokines expressed in the inflamed cells (LPS-treated, as a positive control) before and after treatment with compounds, concerning untreated healthy cells (LPS-unstimulated, as a negative control).

#### 4.1.1. Determination of the Drug’s Optimal Concentrations 

The first and most critical step in the drug-discovery procedure is to define the effective concentration (EC) of a compound used in each experiment. The predicted (theoretical) EC50 was the base for finding the experimental EC values of D5 and ASA values concerning the level of LD50, LC50, and TD50 of compounds. Then, for optimal dosage detection, the experimental EC was used in three doses, including ½ EC50, exact EC50, and 2x EC50 levels, via a dose-determination test on the expression of two types of proinflammatory cytokines (IL-6, and IFN-γ), after a 12h treatment of the inflamed cells. For this purpose, we used two types of ELISA kits, including a IL-6 Human ELISA Kit, a High Sensitivity ELISA Kit (Invitrogen), and an IFN-gamma Human ELISA Kit (Invitrogen), according to their manufacture protocols, with minor modifications. The results (presented in Table 1) indicate that the efficacy level of D5 on IL-6 and INF-γ is a dose-dependent parameter and is correlated to the concentration of the compound. The ANOVA analysis showed significant differences in the levels of INF-γ and IL-6 between various dosages of drugs, and between the positive and negative groups (df = 7, *p* < 0.01, F_crit_ = 2.66 and F value (338.6 for IL-6 and 917.6 for IFN-γ). The post hoc analysis indicated that D5 in a 2xEC dosage exhibited higher efficacy on IL-6 secretion (dF = 4, T_crit_ = 2.8, *p* < 0.01, and T_stat_ = 7.35 and 26.55 compared to EC and 1/2EC, respectively). Although D5 showed dose-dependent inhibition on INF-γ, and D5 demonstrated significant differences between ½ EC and the rest of the doses (dF = 3, T_crit_ = 3.18, *p* < 0.05, and T_stat_ = 14.28), there was no significant difference between EC and 2x EC. However, priority is given to the lowest dose of druggable molecules to minimize the side effects. Therefore, in this study, the optimum EC value of D5 was determined as 0.71 µM of D5 (240 ng/mL) due to the overlapping 240 and 480 ng/mL drug range concentration affecting both IL-6 and IFN-γ expression, respectively. The optimum dose for ASA was estimated to be 52.73 µM of ASA.

#### 4.1.2. Drug Safety 

**Figure 1:**
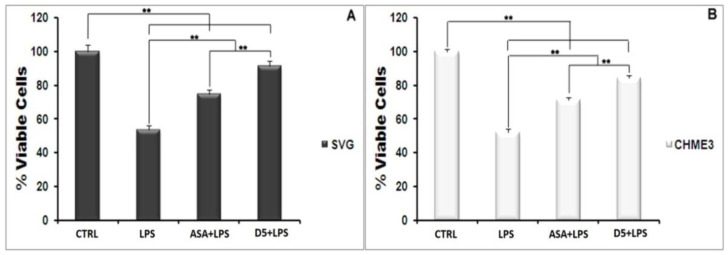
Cytotoxicity Test of D5 (0.71 µM) compared to ASA (52.73 µM), LPS-treated (positive control), and LPS-untreated (negative control) groups. Both SVG astrocytic glial and CHME3 microglial cells were treated with LPS + Drugs for 24 h. Cytotoxicity of D5 was examined using MTT assay (2 mg/mL). The cell viability was measured as % of the amount of formazan formation in the control unstimulated group. (**A**) the D5 cellular toxicity in the selected dose in the SVG cell line; (**B**) the cellular toxicity of D5 in the selected dose in CHME3 cell lines. Data were presented as the mean ± S.D. of four samples in one independent experiment. The data were replicated in three repeated independent experiments. ** *p* < 0.01 probability.

After detecting the effective concentration, it is critical to check the safety of a potent drug molecule for cells and organisms [43]. A MTT assay was used to evaluate the cyto safety of D5 in the selected dose (0.71 µM) and its protection against the cytotoxicity effect of LPS on the glial cells. The ANOVA analysis, at a confidence level of 95%, demonstrated significant differences among groups (df = 3, *p* < 0.01, F_crit_ = 4.07, and F = 409.15), and post hoc analysis showed that D5 significantly enhanced the viability of LPS-stimulated cells, at around 32–33% and 37–38% of the CHME3 and SVG cells with a T_stat_ of 23.48 and 17.16, respectively (df = 4, T_crit_ = 2.78, and *p* < 0.01). For drug competency at the level of cytoprotection efficacy, the level of cell viability in the selected EC was compared between D5-treated and ASA-treated groups using post hoc analysis. The results demonstrated that D5 significantly enhanced the cell viability of both LPS-stimulated CHME3 (T_crit_ = 2.78, *p* < 0.01, df = 4, and T_stat_ = 9.27) and SVG (T_crit_ = 2.78, *p* < 0.01, df = 4, and T_stat_ = 7.81) cell lines, compared to the ASA treatment. Figure 1A,B represent the percentages of cell viability among the trials (D5 and ASA) and control groups (LPS-stimulated and -unstimulated, SVG and CHME3 cells) after 24 h of drug treatments.

#### 4.1.3. Inhibitory Effect of D5 on the iNOS Expression Level and Nitric Oxide Secretion in LPS-Stimulated SVG and CHME3 Cell Lines

**Figure 2:**
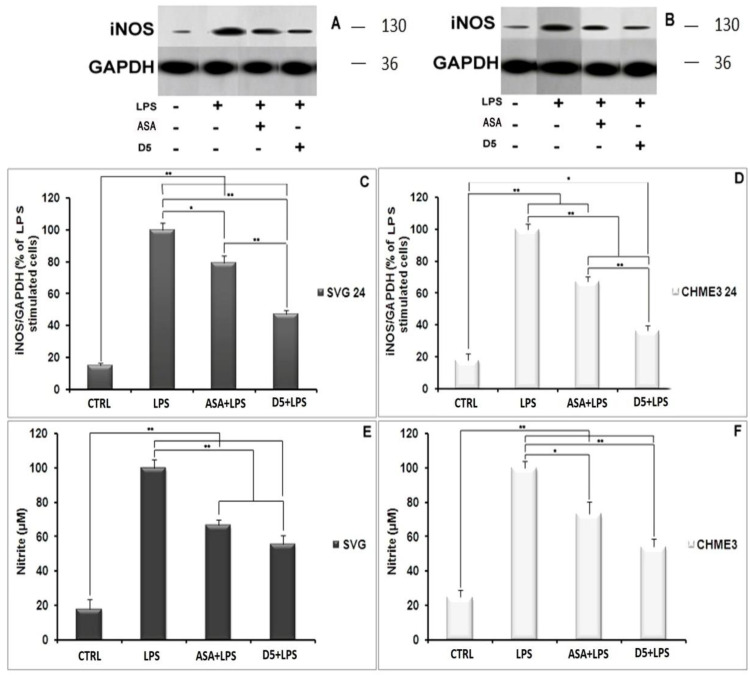
Regulatory impact of D5 on iNOS expression and NO secretion from LPS-stimulated SVG astrocyte and CHME3 microglia cell lines compared to ASA, positive and negative control groups: (**A**,**B**) Figures show the representative images from Western blotting analysis representing the optimum bands of protein assay extracted from SVG (**A**) and CHME3 (**B**); (**C**,**D**) the astrocytic glial (SVG) (**C**) and microglial (CHME3) (**D**) cells were treated with D5 (0.71 µM) and ASA (52.73 µM) for 2 h, and then treated cells were incubated with LPS (100 ng/mL) for 24 h. Thereafter, the total cellular proteins were extracted, and the level of iNOS expression induced by LPS was measured using Western blot analysis. Optical densities of individual bands were quantified using ImageJ software and the obtained data were normalized to the corresponding levels of GAPDH; (**E**,**F**) SVG astrocytic glial and CHME3 microglial cells were treated with D5 (0.71 µM) and ASA (52.73 µM) for 2 h and then incubated with LPS (100 ng/mL) for 24 h. the level of NO secretion (µM) extracted from SVG and CHME3 cells induced by LPS was measured in a culture medium at different concentrations using nitrite assay. The efficacy of D5 on down-regulation of the NO secretion was compared to those of ASA, negative and positive control groups. Data were presented as the mean ± S.D. of four samples in one independent experiment. The data were replicated in three repeated independent experiments. * *p* < 0.05; ** *p* < 0.01 probability.

Nitric oxide secretion among all inflammatory mediators is a well-known indicator of glial cell activation. The synthesis and secretion of NO into the culture medium is a consequence of the expression of iNOS in the activated glial cells [31]. In the present study, the level of iNOS and radical NO secretion in LPS-stimulated microglial and astrocytic glial cells were investigated using Western blot and the Griess-reagent-based nitrite assay before and after treatment withD5. These assays were used to evaluate the regulatory potential of D5 on the inflammatory cascade caused by the glial cell activation (Figure 2A–F). In brief, both glial cell lines were treated for 2 h with 0.71 µM D5 and then stimulated with LPS (100 ng/mL) for 24 h. ANOVA analysis showed significant differences among groups (*p* < 0.01, df = 3, F_crit_ = 4.07; F = 164.79 and 119.06 for NO secretion, and 314.81 and 337.37 for iNOS expression in SVG and CHME3 cell lines, respectively). Post hoc analysis showed that compared to LPS-stimulated cells, both drug-treated groups demonstrated significantly lower iNOS expression levels (df = 4, T_crit_ = 2.78, T_stat_ = 5.48 and 12.42 for the ASA-treated SVG and CHME3 cell lines, with *p* < 0.05 and *p* < 0.01; the mentioned T_crit_ and T_stat_ for the D5-treated SVG cell lineswere3.18 and 17.27, and for the CHME3 cell line were2.78 and 23.20, respectively with *p* < 0.01 for all groups). Additionally, post hoc analysis showed the significant efficacy of D5 on the levels of NO secretion from LPS-stimulated SVG (df = 4, T_crit_ = 2.78, T_stat_ = 10.90, and *p* < 0.01) and LPS-stimulated CHME3 (df = 4, T_crit_ = 2.78, T_stat_ = 13.79, and *p* < 0.01) cell lines treated with D5. ASA demonstrated the same pattern in the alleviation of NO secretion from LPS-stimulated SVG (df = 3, T_crit_ = 3.18, and T_stat_ = 10.23, and *p* < 0.01) and LPS-stimulated CHME3 (df = 3, T_crit_ = 3.18, T_stat_ = 5.77, and *p* < 0.05) cell lines treated with ASA. According to the post hoc analysis, there were no significant differences between D5 and ASA in the inhibition of NO secretion from LPS-stimulated SVG (T_crit_ = 3.18, *p* < 0.05, df = 3, and T_stat_ = 3.43) and CHME3 (T = 3.95, *p* < 0.05, df = 3, and T_crit_ = 3.18) cell lines. Meanwhile, the protein assay data analysis demonstrated that the 2-h pre-treatment of cells with D5 and ASA, before LPS stimulation, significantly alleviated the iNOS expression level in both CHME3 and SVG cell lines after 24 h of incubation. The post hoc analysis showed a significant difference in the iNOS expression level inside the D5-pre-treated LPS-stimulated SVG cells after 24 h of incubation compared to ASA (52.76 ± 2.58% against 20.78 ± 4.72%, T_stat_ = 10.30, *p* > 0.01, T_crit_ = 3.18, and df = 4).The same pattern was found in the level of iNOS expression from D5-pre-treated compared to ASA-pre-treated LPS-stimulated CHME3 cells after 24 h of incubation (63.71 ± 3.24% against 32.66 ± 2.93%, T_stat_ = 12.33, T_crit_ = 2.78, *p* < 0.01, and df = 4). The iNOS mRNA should be checked using RT-PCR to validate the obtained result. 

#### 4.1.4. Inhibitory Effects of D5 on TNF-α and IL-1β Expression in LPS-Stimulated SVG and CHME3 Cell Lines

**Figure 3:**
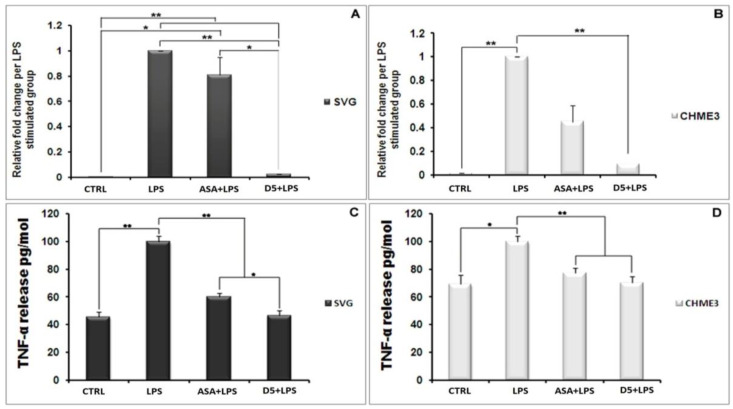
Inhibitory Effects of D5 on *TNF-α* mRNA and protein levels in LPS-Stimulated SVG astrocyte and CHME3 microglia cell lines. SVG astrocytic glial and CHME3 microglial cells were treated with D5 and ASA (0.71 µM and 52.73 µM, respectively) for 2 h, and then incubated with LPS (100 ng/mL) for 24 h. Total cellular mRNA was isolated from SVG (**A**) and CHME3 (**B**) cells and the optical density of individual *TNF-α* mRNA was normalized to the corresponding levels of *GAPDH* mRNA. Amounts of TNF-α released from SVG (**C**) and CHME (**D**) cells into the culture medium were measured using human TNF-α and IL-1β ELISA kits based on the quantitative sandwich enzyme immunosorbent technique. Quantitative analyses were shown as % or fold increases in the control unstimulated group. Data are presented as the mean ± S.D. of four samples in one independent experiment. The data were replicated in three repeated independent experiments. One-way ANOVA followed by post hoc analysis was performed for the comparison of the mean values of the level of *TNF-α* mRNA and protein expressed in the D5-treated group and the rest of the groups. * *p* < 0.05; ** *p* < 0.01 probability.

In an inflammatory scenario, proinflammatory cytokines are expressed as the inter- and intra-cellular signaling messengers [44]. Therefore, the inhibition of proinflammatory cytokines is a promising treatment for most disorders caused by inflammation. With this in mind, the effect of D5 was tested on the expression level of proinflammatory cytokines in the LPS-stimulated CHME3 and SVG cells with a concentration of 100 ng/mL, in the presence or absence of D5 (0.71 µM) and ASA (52.73 µM),for 24 h. The cells were pre-treated with D5 and ASA for 2h before LPS stimulation. The levels of *TNF-α* mRNA transcription (Figure 3A,B), and TNF-α protein secretion into the culture medium (Figure 3C,D) were assayed using qPCR and ELISA assays. The ANOVA analysis showed significant differences in the level of mRNA and protein secretion of TNF-α among the groups of both cell lines (df = 3, F_crit_ = 4.066, *p* < 0.01; F = 163.260 and 115.387 for mRNA inhibition inside, and 176.945 and 27.845 for protein secretion from SVG and CHME3 cells, respectively). The post hoc study demonstrated that both mRNA and protein secretion of TNF-α were remarkably increased by around 285.747 and 2.189 in the LPS-stimulated SVG (df = 2, T_crit_ = 4.303, *p* < 0.01, T_stat_ = 16898.9; df = 4, T_crit_ = 2.776, *p* < 0.01, T_stat_ = 18.817), while in the LPS-stimulated CHME3 cells;and the rates of mRNA and protein secretion of TNF-α were up-regulated by around 69.610 and 1.443 (df = 2, T_crit_ = 4.303, *p* < 0.01, T_stat_ = 577.191; df = 3, T_crit_ = 3.182, *p* < 0.05, T_stat_ = 6.986) compared with the negative control groups. The post hoc analysis of the drug’s efficacy demonstrated that D5 significantly inhibited *TNF-α* transcription and cytokine secretion by 97.526 ± 0.088% and by 53.263 ± 3.516% in the LPS-stimulated SVG (df = 2, T_crit_ = 4.303, *p* < 0.01, T_stat_ = 1925.752; df = 4, T_crit_ = 2.777, *p* < 0.01, T_stat_ = 17.801). Meanwhile, D5 significantly inhibited *TNF-α* transcription and cytokine secretion in the LPS-stimulated CHME3 cells by around 90.45 ± 0.027% and 29.589 ± 4.137% (df = 2, T_crit_ = 4.303, *p* < 0.01, T_stat_ = 57.767; df = 4, T_crit_ = 2.777, *p* < 0.01, T_stat_ = 8.760) compared to the LPS-stimulated group. Although ASA showed noticeable inhibitory function on the level of *TNF-α* mRNA expression and TNF-α pro-inflammatory cytokine—by around 1.24 and 1.66 in the LPS-stimulated SVG and 2.228 and 1.294 in the LPS-stimulated CHME3 cells, respectively—in comparison with LPS-stimulated group, the post hoc analysis demonstrated significantly lower inhibitory efficacy of ASA compared to that of D5 on the *TNF-α* mRNA transcription in LPS-stimulated SVG (T_stat_ = 9.610, T_crit_ = 4.303, *p* < 0.05, and df = 2). The comparison of the potential inhibitory function of D5 and ASA for the secretion of TNF-α in media showed that D5 demonstrated significantly higher efficacy than ASA to reduce the level of TNF-α secreted from LPS-stimulated SVG cells (T_stat_ = 5.371, T_crit_ = 2.777, *p* < 0.05, and df = 4). 

**Figure 4:**
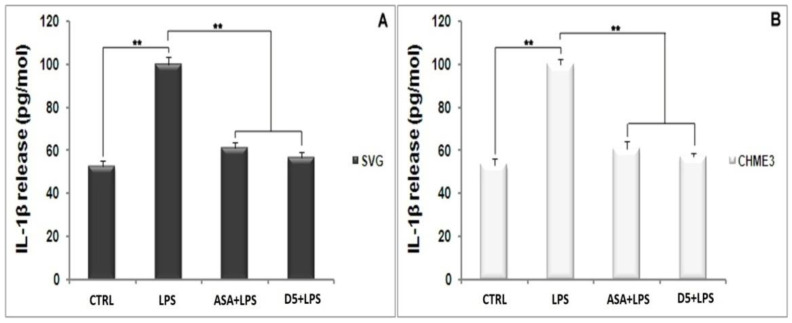
Inhibitory Effects of D5 on IL-1β production in LPS-Stimulated astrocyte (SVG) and microglia (CHME3) cell lines. Astrocytic glial (SVG) and microglial (CHME3) cells were treated with D5 and ASA (0.71 µM and 52.73 µM, respectively) for 2 h, and then incubated with LPS (100 ng/mL) for 24 h. Amounts of IL-1β released from SVG (**A**) and CHME3 (**B**) cells into the culture medium were measured using a human IL-1β ELISA kit based on the quantitative sandwich enzyme immunosorbent technique. Quantitative analyses were shown as % or fold increases in the control unstimulated group. Data are presented as the mean ± S.D. of four samples in one independent experiment. The data were replicated in three repeated independent experiments. ** *p* < 0.01 probability.

The same pattern occurs in the level of IL-1β secretion from the LPS-stimulated SVG and CHME3 cells (Figure 4A,B). The post hoc analysis showed that the IL-1β secretion was significantly up-regulated by LPS stimulation by 190.191 ± 13.286% and 187.619 ± 5.909% compared to the unstimulated group (T_crit_ = 3.182, *p* < 0.01, T_stat_ = 19.246, df = 3; T_crit_ = 2.777, *p* < 0.01, T_stat_ = 20.869, and df = 4). However, the mentioned levels in the D5-pre-treated LPS-stimulated SVG and CHME3 cells markedly dropped by 43.304 ± 5.486% and 42.835 ± 1.606%, respectively. The results showed that ASA could suppress IL-1β secretion from LPS-stimulated SVG and CHME3 cells, at about 38.839 ± 4.397% and 39.177 ± 1.056%, respectively. The comparative study on the efficacies of both ASA and D5 demonstrated that there is no significant difference between the inhibitory function of mentioned drugs on the level of IL-1β secretion from both LPS-stimulated SVG and CHME3 cells. For the validation of our obtained results, itis necessary to check the mRNA level using RT-PCR.

#### 4.1.5. Up-Regulation of PPAR-γ Receptor Expression by D5 in LPS-Stimulated SVG and CHME3 Cell Lines

**Figure 5:**
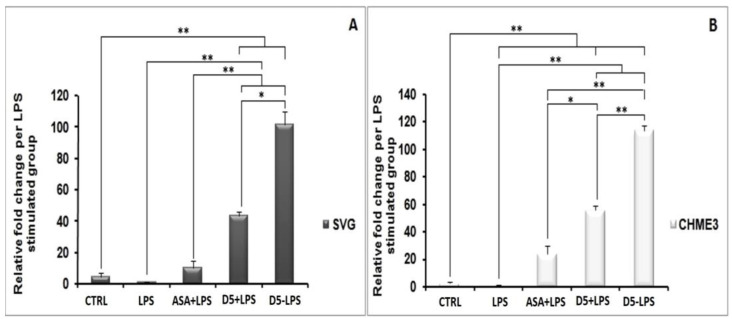
Up-regulation of PPAR-γ receptor expression by D5 in LPS-stimulated astrocyte (SVG) and microglia (CHME3) cell lines. (**A**) Astrocytic glial (SVG) and (**B**) microglial (CHME3) cells were treated with D5 and ASA (0.71 µM and 52.73 µM, respectively) for 2 h, and then incubated with LPS (100 ng/mL) for 24 h. Total cellular mRNA was isolated and the normalization of the optical density of individual *PPAR-γ* mRNA was performed usingthe corresponding levels of *GAPDH* mRNA. Data are presented as the number of fold changes based on the level of *PPAR-γ* mRNA expression in the positive control groups of both cell lines. Data are presented as the mean ± S.D. of four samples in one independent experiment. The data were replicated in three repeated independent experiments. * *p* < 0.05; ** *p* < 0.01 probability.

In the brain, PPAR-γ actsas an anti-inflammatory messenger and expresses in several cell types, including neuronal cells and neuroimmune cells such as microglia, astrocytes, and oligodendrocytes [45]. Therefore, PPAR-γ expression stimulants not only accelerate the inhibition of NF-κB DNA-binding activity, but also suppress the acute phase of neuroinflammation mediated by IL-6; this suggests that it could be a promising therapeutic strategy in most brain disorders [46]. In the present study, the effect of D5 on *PPAR-γ* mRNA expression was checked in LPS-stimulated CHME3 and SVG cells for 24 h. The cells were pre-treated with D5 (0.71 µM) for 2h before LPS stimulation. The result indicated that LPS stimulation reduced the level of *PPAR-γ* mRNA expression in both SVG and CHME3 cells by around 4.515 and 2.324compared with the negative control (CTRL) groups. However, there was no significant difference observed between the LPS-stimulated and CTRL groups of each cell line. The treatment of LPS-stimulated cells with D5 and ASA remarkably retrieved the level of the *PPAR-γ* mRNA expression. The ANOVA analysis of the obtained data demonstrated significant differences among groups (df = 4, F_crit_ = 3.478, *p* < 0.01, and F = 275.45 for SVG and 591.71 for CHME3). The post hoc analysis indicated that *PPAR-γ* mRNA expression (Figure 5A,B) was significantly up-regulated by the D5 treatment of LPS-stimulated SVG and CHME3 cell groups by around 4313.11 + 283.57%, and 5562.395 + 288.788% (df = 2, *p* < 0.01, T_crit_ = 4.303;T_stat_ = 25.735 for SVG and 32.761 for CHME3). In the ASA-treated group, the level of PPAR-γ mRNA expression was increased by up to 1046.06 ± 392.866% and 2377.417 ± 558.021% compared with LPS-stimulated control groups. However, the post hoc analysis did not show any significant changes between ASA-treated and untreated LPS-stimulated SVG and CHME3 cells. The comparison study using post hoc analysis showed that D5 demonstrated significantly higher efficacy than ASA, with regard to its effective EC, to induce cellular PPAR-γ mRNA expression in the LPS-stimulated SVG cells (T_crit_ = 2.776, *p* < 0.01, df = 4, and T_stat_ = 11.680), while in CHME3, the difference was found at aconfidence level of 95% (*p* < 0.05) (T_crit_ = 3.182, T_stat_ = 8.780, and df = 3). The treatment negative control with D5 indicated that D5 significantly up-regulated the level of *PPAR-γ* mRNA compared to the positive control groups of both cell lines treated with D5, by around 235.23 ± 19.23%, with *p* < 0.01 (df = 2, and T_crit_ = 4.302) for SVG and 204 ± 6.97% (df = 4, and T_crit_ = 2.78) for CHME3. The results showed that D5 acted as a PPAR-γ agonist and significantly up-regulated the PPAR-γ mRNA level in both cell lines in the presence or absence of LPS (Figure 5). To investigate the drug’s efficacy on the level of PPAR-γ expression, Western blot analysis is necessary.

#### 4.1.6. Inhibitory Effects of D5 on NF-κBSignaling in LPS-Stimulated SVG and CHME3 Cell Lines

**Figure 6:**
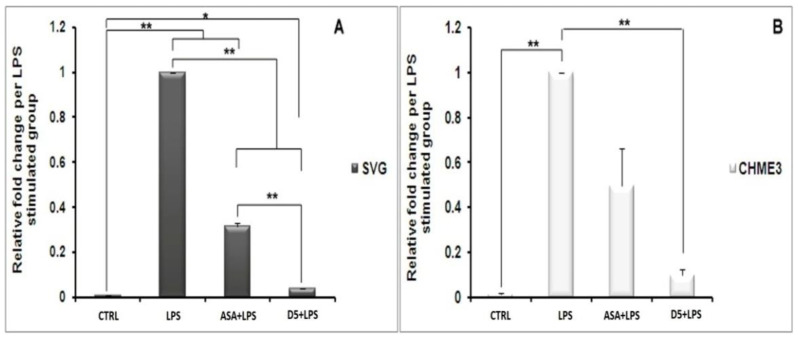
Regulatory effects of D5 on IKK-β mRNA expression in LPS-Stimulated astrocyte (SVG ) and microglia (CHME3 ) cell lines. (**A**) Astrocytic glial (SVG) and (**B**) microglial (CHME3) cells were treated with D5 and ASA (0.71 µM and 52.73 µM, respectively) for 2 h, and then incubated with LPS (100 ng/mL) for 24 h.Total cellular mRNA was isolated and the normalization of the optical density of individual *IKK-β* mRNA was performed using the corresponding levels of GAPDH mRNA. Data are presented as the number of fold changes based on the level of *IKK-β* mRNA expression in the positive control groups of both cell lines. Data are presented as the mean ± S.D. of four samples in one independent experiment. The data were replicated in three repeated independent experiments. * *p* < 0.05; ** *p* < 0.01 probability.

**Figure 7:**
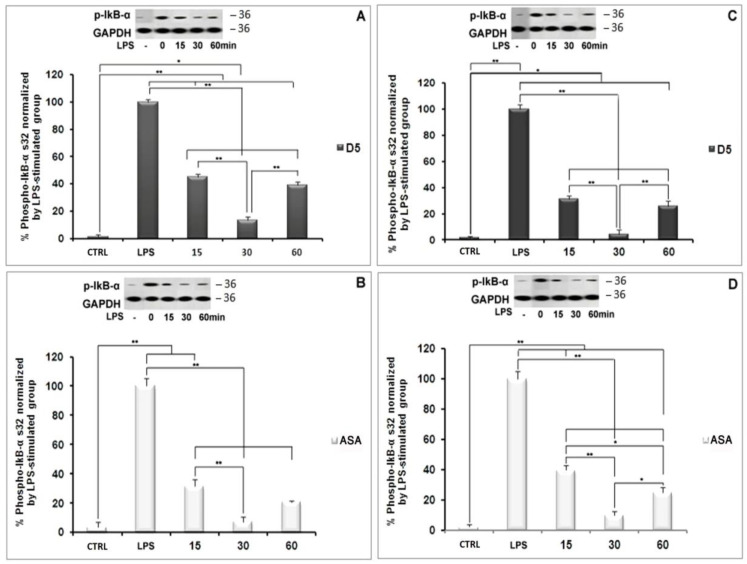
**NF-κB silencing using D5 treatment of LPS-Stimulated astrocyte** (**SVG**) **and microglia** (**CHME3**) **cell lines.** Astrocytic glial (SVG) and microglial (CHME3) cells were pretreated with D5 (**A**,**C**, respectively) and ASA (**B**,**D**, respectively) for 24 h before LPS stimulation (100 ng/mL) at the indicated time points (0 (LPS), 15, 30, and 60 min). Total cellular proteins were extracted and IκB-α (phospho-IκB-α S32) protein levels in SVG and CHME3 cells were estimated to investigate LPS-induced NF-κB activation in the cytosol of glial cells using Western blot analysis. Optical densities of individual protein bands were normalized to the corresponding levels of GAPDH. The figures represent the best protein bands and their quantitative analyses. Groups were compared using the percentages of the positive control groups of both cell lines at time 0 (drug-untreated LPS-stimulated cells). Data are presented as the mean ± S.D. of four samples in one independent experiment. The data were replicated in three repeated independent experiments. * *p* < 0.05; ** *p* < 0.01 probability.

**Figure 8:**
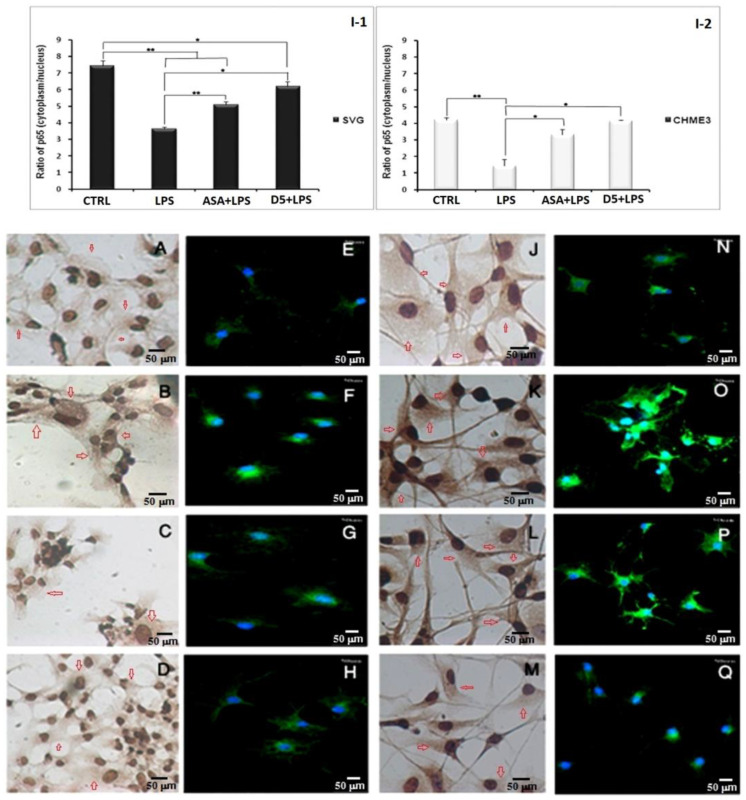
In situ study using immunocytochemistry and immunofluorescence assay to investigate the impact of D5 treatment on silencing NF-κB signaling pathway in LPS-Stimulated astrocyte (SVG) and microglia (CHME3) cell lines:(**A**–**D**,**J**–**M**) Representative images of hematoxylin/DAB immunocytochemistry study of the D5 and ASA treatments roles on silencing NF-κB signaling pathway in LPS-Stimulated astrocyte (SVG) and microglia (CHME3) cell lines. Images are shown at 400-fold magnification. The in situ study demonstrated the impact of D5 compared to ASA on the decomposition of NF-κB complex, induced by LPS in the cytosol and subcellular localization of phospho-IκB-α S32. DAB-labeled HRP against Anti-Phospho-I B alpha (S32) Rabbit Monoclonal Antibody was used to produce brown dots in the peripheral cytoplasm to nuclei (depicted by red arrows in the ICC images), counterstained with Hematoxylin to form dark-blue-to-black stains in control groups of astrocyte (**A**) and microglia cells (**J**). In the LPS-stimulated astrocyte (**B**) and microglia (**K**) groups, phospho-I B alpha(S32) subunit immunoreactivities were shown in the cytosol. ASA and D4 treatments significantly protect the IκB-α subunit from phosphorylation and keep the NF-κB complex, in the cytosol of astrocytic glial and microglial cells, in its inactive form ((**G**,**H**) and (**P**,**Q**), respectively). The level of activated NF-κB-p65 inside the nucleus was measured using a fluorescent-tagged mouse monoclonal NF-κB p65 antibody. The high level of fluorescent signal in the nucleolus of cells is an indicator of the phosphorylation of the IκB-α subunit, breakage of NF-κB complex in the cytosol, activation, translocation of the NF-κB into the nucleus, and binding to chromosomal DNA, resulting in the gene expression of various pro-inflammatory cytokines. The immunofluorescence assay of the activated NF-κB-p65 nuclear translocation in both drug-pre-treated SVG and CHME3 cells stimulated with LPS was performed using a 5 µg/mL mouse monoclonal NF-κB p65 antibody (Thermo-Fisher, Poland) and 10 µg/mL goat anti-mouse IgG Alexa Fluor488 (Thermo-Fisher, Poland) as the primary and secondary antibodies, respectively. The fluorescent signals in samples were obtained from the complex of NF-κB p65 antibody-IgG Alexa Flour488 (the green dots in cytoplasm and nuclei), and nuclear counter stain DAPI (the blue channel signals in the control microglia and astrocyte groups) ((**E**–**H**) for SVG and (**N**–**Q**) for CHME3 cells). It is observed that 60 min after LPS stimulation of pre-treated CHME3 and SVG cells, the density of the green signal increased in the nuclei of cells, suggesting the translocation of NF-κB to the nucleus. The images were quantified using Cell profiler software, and data are presented as the ratio of the green fluorescent density between cytoplasm and nuclei of cells (**I-1**,**I-2**). * *p* < 0.05; ** *p* < 0.01 probability. The image scale is 50 µm.

NF-κB nuclear translocation is a well-known signaling pathway upstream of proinflammatory cytokine and chemokine expression and secretion [47]. The NF-κB complex in its inactive form is linked to the inhibitory subunit IκB in the cytosol of glial cells, including microglia and astrocytes [48]. Following the deterioration of IκB, NF-κB is activated and migrates from the cytosol to the nucleus, where it binds to genomic DNA and contributes to the expression of various cytokines, such as TNF-α, IL-1β, and iNOS [49,50]. The deterioration of the inhibitory protein IκB occurs following the phosphorylation process by IκB kinase (IKK) and the ubiquitination process [49]. Due to the critical role of the NF-κB pathway in the inflammatory process, we investigated the inhibitory potential of D5 on the nuclear translocation process of the NF-κB complex. We evaluated the expression levels of the *IKK-β* gene, the cytoplasmic level of radical phospho-IκB, the nucleic level of p65 protein; moreover, to estimate the rate of the phosphorylation process, we evaluated the expression levels of the NF-κB complex breakage and activation in the cytosol, and the rate of the nuclear translocation of activated NF-κB, respectively (Figure 6, Figure 7 and Figure 8). After 2 h of treatment with D5 and ASA, the SVG and CHME3 cells were stimulated with LPS (100 ng/mL) for 24 h; then, the total cellular RNA was extracted and used for evaluating changes in the level of *IKK-β* gene expression using RT-PCR, with a primer specific for *IKK-β* transcription mRNA. The results indicated that the mRNA level of *IKK-β* was significantly decreased in D5- and ASA-pre-treated LPS-stimulated SVG and CHME3 cells compared to drug-untreated LPS-stimulated cells. The ANOVA analysis indicated that there are significant differences between groups (df = 3, F_crit_ = 4.066, *p* < 0.01; F = 8799.572 for SVG and 88.694 for CHME3). Post hoc analysis showed that D5 significantly reduced the level of *IKK-β* transcription mRNA by around 96.35 ± 0.29% and 90.21 ± 2.76% in LPS-stimulated SVG and CHME3, respectively (df = 2, T_crit_ = 4.303, *p* < 0.01; T_stat_ = 566.122 for SVG and 56.563 for CHME3). The post hoc analysis indicated that D5 inhibited the gene expression of IKK-β at a remarkably higher level than ASA, with redundancy levels around 27.58 ± 1.95% for SVG (*p* < 0.01, df = 2, T_crit_ = 4.303, and T_stat_ = 28.06), while there was no significant difference in CHME3 cell line. Figure 6 represents the percentage of the change in the mRNA level of *IKK-β* in D5- and ASA-pre-treated LPS-stimulated SVG and CHME3 compared to the positive controls (LPS-stimulated SVG, and CHME3 cells) 24 h after treatment. Suppression of the IKK-β enzyme leads to protection of the IκB-α subunit from phosphorylation at serine 32 and 36 and deterioration, resulting in silencing of the NF-κB complex in the cytosol [51]. To investigate the consequence of *IKK-β* gene inhibition on IκB-α phosphorylation, we evaluated the level of radical phosphorylated IκB-α in cytosol using Western blot, and tracked the p65 subunit of active NF-κB in the nucleus using the immunocytochemistry method. Further studies are necessary to investigate the role and impact of druggable compounds on IKK-β enzymatic activity and protein levels.

CHME3 and SVG cells were pre-treated with D5 2 h before stimulation by LPS (100 ng/mL) for the designated times (15, 30, and 60 min). The controls (positive and negative) were set for each experiment. Then, the total protein was extracted and exposed to 10% SDS-PAGE. Western blot was used to evaluate the level of radical phospho-IκB using rabbit anti-IκB-phospho S32 antibody. The ANOVA analysis showed significant differences among the treated group at various time points and the controls. The post hoc analysis demonstrated that the level of phospho-IκB-α protein was significantly decreased in the LPS-stimulated SVG cells, when treated with D5 for 15 min, by 54.95 ± 1.90% (df = 4, T_stat_ = 32.64, *p* < 0.01, and T_crit_ = 2.776), and reached the maximum inhibitory level up to 86.59 ± 2.51% at the 30 min time point (df = 4, T_stat_ = 44.79, *p* < 0.01, and T_crit_ = 2.776). The result showed a level of correlation between the time and the IκB subunit degradation induced by LPS. However, the level of IκB-α considerably recovered to 61.19 ± 2.78% (df = 4, T_stat_ = 29.84, *p* < 0.01, and T_crit_ = 2.776) after one hour compared to the LPS-stimulated group (Figure 7A). The post hoc analysis showed the same pattern in CHME3 cells when pre-treated with D5. The level of cytoplasmic radical phospho-IκB was significantly mitigated after 15 min, and the inhibitory impact of D5 was maximized at 30 min from 68.66 ± 2.462%, to 95.59 ± 3.31% (df = 4, *p* < 0.01, T_crit_ = 2.776;T_stat_ = 30.19 for 15 min and 36.60 for 30 min time points); however, it recovered at the 60 min time point, reaching 74.25 ± 3.83%, compared to the LPS-stimulated group (df = 4, T_stat_ = 26.18, *p* < 0.01, and T_crit_ = 2.776) (Figure 7C). The compression between the inhibitory efficacy of ASA and D5 using ANOVA analysis followed by post hoc revealed that D5 was a significantly better silencer of the NF-κB complex than ASA at 15 min, with *p* < 0.05 (df = 4, T_crit_ = 3.18, T_stat_ = 4.82) and at 60 min, with *p* < 0.01 (T_crit_ = 4.30, df = 2, and T_stat_ = 10.80). It suggested that D5 can protect the IκB-α subunit from phosphorylation and degradation in the SVG cells at all time points (Figure 7B). In contrast, D5 in CHME3 cells did not show any more significant efficacy on the level of radical IκB-α in the cytosol than ASA, except for at the 15 min time point. D5 showed more significant inhibitory function at the 15 min time point (with *p* < 0.05, df = 4, T_crit_ = 2.776, and T_stat_ = 3.599) on the level of cytoplasmic radical phospho-IκB-α in CHME3 cells than ASA (Figure 7D).

We checked the changes in the level of radical IκB-α in the cytosol using an in situ technique called the hematoxylin-based immunocytochemistry (ICC) assay. The assay was used to visually evaluate the rate of activated NF-κB nuclear translocation using rabbit anti-phospho-IκB-α (S32) and HRP as the primary and secondary antibodies. In the mentioned assay, if there is a high density of brown color in the cytosol of cells, this indicates the phosphorylation of the IκB-α subunit at the Ser32 residue, resulting in the translocation of activated NF-κB into the nucleus. In contrast, a low density of brown color in the cytosol indicates ahigh level of inactive NF-κB in the cytosol and a low rate of translocation. Our results showed that D5 significantly reduced the density of brown color in the cytosol of cells, indicating the alleviation of radical phospho-IκB-α in the cytosol. To capture microscopic images of the immunostaining against phospho-IκB-α (S32), an Olympus IX70 microscope was used at a magnification of 400 (Figure 8A–D for SVG and J–M for CHME3 cells). However, due to the low quality of the pictures, the quantification of the images was not possible, so we just used them for the qualitative representation of NF-κB behavior in response to the drug treatments. To compensate for the mentioned problem, we conducted another immunocytochemistry study using immunofluorescence staining, using a fluorescent-tagged mouse monoclonal antibody against the p65 subunit of the activated NF-κB [52]. All experimental conditions and the incubation time scales were kept constant, the same as previous non-fluorescent immune peroxidase staining ICC, as a complementary quantitative study. The ratio of fluorescent signals between the cytosol and nucleus of a cell can quantitatively determine the translocation level of activated NF-κB [53]. The high level of fluorescent signal in the nucleolus of cells is an indicator of the phosphorylation of the IκB-α subunit, breakage of the NF-κB complex in the cytosol, activation, translocation of NF-κB into the nucleus, and binding to chromosomal DNA, resulting in the gene expression of various pro-inflammatory cytokines. The obtained data from fluorescent ICC may be used to interpret the level of radical phospho-IκB-α (S32) found in the cytosol of our previous non-fluorescent ICC. The combination of data obtained from non-fluorescent and fluorescent ICCs can localize the radical phospho-IκB-α and activated NF-κB p65 subunits in the cytosol and nucleus. It can verify the level of activation and translocation of activated NF-κB after LPS stimulation and drug treatments, and can confirm the efficacy of drugs to inhibit or down-regulate the NF-κB pathway during the inflammatory process. 

The immunofluorescence assay of the activated NF-κB-p65 nuclear translocation in both drug-pre-treated SVG and CHME3 cells stimulated with LPS was performed using a 5 µg/mL mouse monoclonal NF-κB p65 antibody (Thermo-Fisher, Poland) and10 µg/mL goat anti-mouse IgG Alexa Fluor488 (Thermo-Fisher, Poland) as the primary and secondary antibodies, respectively. The fluorescent signals in the samples were obtained from the complex of NF-κB p65 antibody-IgG Alexa Flour488 (the green dots in the cytoplasm and nuclei), and nuclear counterstain DAPI (the blue channel signals in the control microglia and astrocyte groups) (Figure 8E–H for SVG and N–Q for CHME3 cells). It was observed that 60 min after LPS stimulation of the pre-treated CHME3 and SVG cells, the density of the green signal increased in the nuclei of the cells, suggesting the translocation of NF-κB to the nucleus. The images were quantified using Cellprofiler software, and the data were presented as the ratio of the green fluorescent density between cytoplasm and nuclei of cells (Figure 8I(1,2)). The ANOVA followed by post hoc analysis of the quantitative data indicated that D5 treatment remarkably inhibited the nuclear translocation of the activated NF-κB p65 subunit by around 1.69 ± 0.065 and 3.056 ± 0.948 in SVG and CHME3, respectively, compared to the LPS-stimulated groups (df = 2, *p* < 0.05, T_crit_ = 4.303; T_stat_ = 13.870 for SVG and 12.260 for CHME3 cells) (Figure 8H,Q). However, the post hoc analysis demonstrated no significant difference between the efficacy of D5 and ASA on the level of nuclear translocation of activated NF-κB, in both LPS-stimulated SVG and CHME3 cells.

### 4.2. Comparison of the Anti-Neuroinflammatory Potential of Methylenedioxy (D5) and Hydroxymethyl (D4) Piperamide Derivatives 

To increase the octal/water fraction coefficient and improve the absorption level through MDCK cells, and obtain barriers for the orally administered drug of our previous piperamide derivative (D4), we modified the hydroxymethyl group with a methylenedioxy group (D5). This alteration may improve drug availability to the CNS through the blood–brain barrier [54].

Following the investigation of the anti-neuroinflammatory potential of D5, it is important to check whether or not the structural modification improved the physicochemical properties and drug efficacy of the novel pharmacophore (D5) compared to previous models (D4) in a series of side-by-side experiments under the same experimental conditions. Hence, we kept all experimental conditions constant for both compounds—including the time of drug exposure, the types of cell lines, the passage number of cell lines, the laboratory conditions, the day of in vitro testing, the level and type of media, the incubation periods, all the solvents for the in vitro and force-field testing, the type of receptor, the binding site of an enzyme, the version of the software, the number of poses, and the biological pH for in silico studies—to perform a valid efficacy comparison of both piperamide derivatives.

Next, we performed several statistical comparisons of the physicochemical and pharmacological properties of D5 with its prototype, D4. The pairwise two-tailed *t*-test analysis was performed on the efficacy and physicochemical properties of both analogs obtained from several in silico, in vitro, and in situ studies, with the same experimental conditions.

#### 4.2.1. In Silico Comparison between Pharmacological Properties of D5 and D4

Pharmacokinetics (PK) is the study of what the body does to a drug, and pharmacodynamics (PD) is the study of what a drug does to the body. Both PK and PD refer to various terms. The former points to the timeline of the drug’s absorption, bioavailability, distribution, metabolism, and excretion from the body, while the latter refers to the ligand-receptor bindings, post-receptor effects, and chemical interactions, along with the toxicological properties of compounds.

- Comparison of the physicochemical and toxicological properties of both compounds

**Figure 9:**
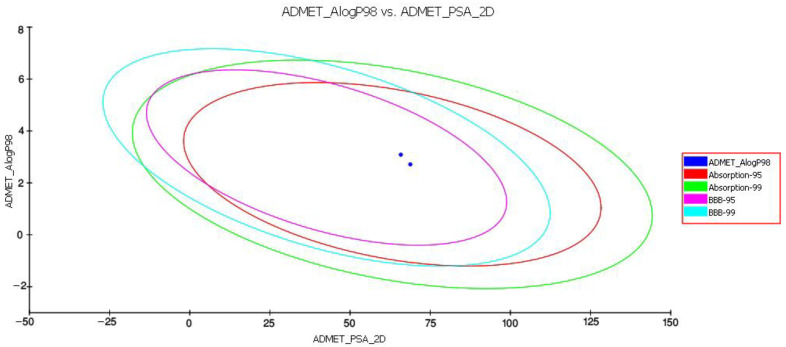
**Plot of polar surface area** (**PSA**) **vs. LogP for D5 and D4**. Data show the 95% and 99% confidence limit ellipses corresponding to the blood–brain barrier and intestinal absorption models.

The PK investigation of D4 and D5 was performed using the Qikprop approach of Schrodinger suite 2018, to evaluate the impact of functional-group alteration on the physiochemical and pharmacological profiling of piperamides. The results indicated that replacing the hydroxymethyl in D4 with a methylenedioxy group in D5 improved some physicochemical properties such as CNS, QPlogPo/w, QPlogHERG, QPlogBB, QPPMDCK, QPlogKp, QPlogKhsa, QPlogS, and QPPCaco; however, functional-group alteration has no effect on the toxicity profile of piperamides. The parameters of the rule of three and the oral absorption comparison of both compounds demonstrated [54] that although all the parameters fell within the recommended ranges, functional-group alteration could improve the level of drug absorption (Figure 9).

The in silico data analysis showed that the functional-group modification increased the level of compound absorption from MDCK cells and the gut–blood barrier. Additionally, alteration of the functional group in D5 reduced compound toxicity for the HERG channel. Table 2 contains the physiochemical properties and their acceptable ranges along with the toxicity profile of both D4 and D5, computed using the Qikprop module of Schrodinger suite 2018 and TOPKAT approach of Accelrys discovery studio 2.5.

b. Comparison of the pharmacodynamic properties of D4 and D5 against IKK-β enzyme

**Figure 10:**
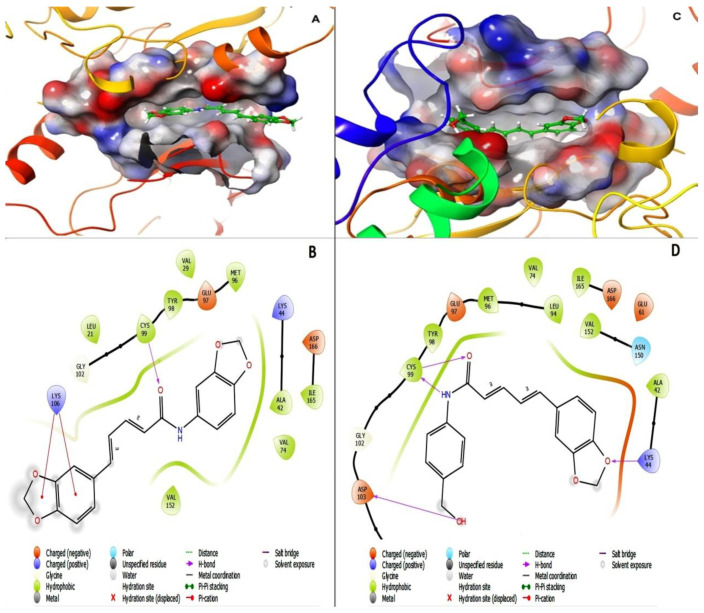
**Interaction of D5 and D4 with the active site of the IKK-β enzyme** (**PDB ID: 4KIK**): The 3D structures of D5 (**A**) and D4 (**C**) in the active site of IKK-B were generated using the visualization approach of Schrodinger site 2018. D4 interacts with the active site of IKK-β with an affinity value of −7.29 kcal/mol and binding energy of −64.74 kcal/mol, with 4 hydrogen bonds to LYS104, CYS99, and ASP103, while D5 shows a G-score of −6.90 kcal/mol and binding energy of −51.15 kcal/mol, with a single hydrogen bond to CYS99 and 2 Pi-cation bonds. The data show that the affinity of piperamides for the binding packet of the IKK-β enzyme could not be improved. The figure shows the 2D structures of D5 (**B**) and D4 (**D**) in the active site of IKK-β, with active site residues including TYR23, GLY22, LEU21, ASP103, GLY102, CYS99, TYR98, GLU97, MET96, VAL74, ALA42, VAL29, VAL152, ASN150, ILE165, ASP166, GLU149, LYS147, and ASP145. Red lines indicate hydrogen bonds.

The level of binding affinities and their respective energies of D4 and D5 for the active site of the IKK-β enzyme were compared to determine the best functional-group modification for improving the efficacy and druggability of piperine. The result indicated that D4 had an affinity value of −7.29 kcal/mol and a binding energy of −64.74 kcal/mol with four hydrogen bonds to LYS104, CYS99, and ASP103, while D5 showed a G-score of −6.90 kcal/mol and a binding energy of−51.15 kcal/mol, with a single hydrogen bond to CYS99 and two Pi-cation bonds. The data showed that the affinity of piperamides for the binding packet of the IKK-β enzyme could not be improved. In addition, the functional-group alteration decreased the stability of the inhibitor inside the binding pocket of the enzyme by reducing the number of H-bonds. However, the other force-fields, such as the Pi-cation bond between inhibitor and binding pocket residues, may compensate for the reduction in H-bonds; however, it will not be able to improve the stability of D5 in the binding pocket of the receptor compared to D4. Figure 10 represents the 2D and 3D structures of ligands bound to the active site of the IKK-β enzyme, and shows the number of H-bonds between compounds and residues in the binding pocket of the receptor. In Figure 10, section A represents the 3D structure of the D5-IKK-β complex, section C shows the 3D structure of the D4-IKK-β complex, and sections B and C depict the 2D structures of D5 and D4 compounds inside the active site of the IKK-β enzyme, respectively. The purple indicates the H-bond between ligands and receptors, and the red line represents the Pi-cation interaction between compounds and enzymes.

#### 4.2.2. In Vitro Comparison of the Druggability Potential of Methylenedioxy and Hydroxymethyl Piperamide Derivatives on LPS-Induced inflammation in the Glial Cells

The pairwise Student’s *t*-test was performed to compare the obtained results from various in vitro studies examining the efficacy of methylenedioxy piperamide and hydroxymethyl piperamide on the inflammatory pathways in glial cells. The level of significant differences between groups was determined to be *p* < 0.05. The in vitro tests consisted of assessing the level of cytoprotection using an MTT assay, NO quantification using Griess reagent, protein secretion (iNOS and phospho-IκB) using Western blot analysis, cytokine secretion (TNF-α, IL-6, INF-γ, and IL-1β) using an Enzyme-Linked Immunosorbent Assay (ELISA), and cytokine and enzyme gene expression (IKK-β, PPAR-γ, and TNF-α) using quantitative real-time PCR. 

The *t*-test analysis did not show any significant differences in the level of cytosafety for either of the analogs. In contrast, D5 demonstrated remarkably higher efficacy than D4 in reducing the levels of NO and iNOS in both SVG and CHME3 cells. D5 reduced the level of NO by around 1.3 ± 0.19 in SVG cells, with *p* < 0.05 (df = 4, T_crit_ = 2.776, and T_stat_ = 3.617) and 1.62 ± 0.18 times in CHME3 cells, with *p* < 0.01 (df = 2, T_crit_ = 4.303, and T_stat_ = 12.727), in contrast to D4 (Table 3). Additionally, D5 suppressed iNOS secretion from SVG cells at a significantly higher level than D4, by around 1.26 ± 0.15, with *p* < 0.05 (df = 3, T_crit_ = 3.182, and T_stat_ = 4.458). The comparison study showed a similar pattern in the release of iNOS from CHME3 treated with D5 and D4. D5 significantly reduced the secretion of iNOS from CHME3 cells by around 2.19 ± 0.07, with *p* < 0.01 (df = 4, T_crit_ = 2.776, and T_stat_ = 12.893) compared with D4 (Table 3). The efficacies of D5 and D4 compounds on the protection of the phospho-IκB-α subunit from phosphorylation by IKK-β were studied, and the comparison results indicated that, except for the 60 and 15 min time points in SVG and CHME3 cells, respectively, there were no significant differences observed among the D5 and D4 efficacies on IκB-α protection among the treated groups at the various time points. According to the *t*-test comparison, D4 showed better efficacy on IκB-α protection against phosphorylation than D5 in SVG cells at the 60 min time point by around 1.78 ± 0.26, with *p* < 0.01 (df = 3, T_crit_ = 3.182, and T_stat_ = 8.816); meanwhile, in CHME3 cells, D5 showed more remarkable efficacy on IκB-α protection at the 15 min time point than D4 by around 1.245 ± 0.003, with *p* < 0.05 (df = 4, T_crit_ = 2.776, and T_stat_ = 3.417) (Table 3).

The comparison study on the efficacies of D5 and D4 on cytokine expression and secretion showed that D5 could more significantly reduce the level of TNF-α in SVG cells after 24 h than D4 by about 1.259 ± 0.089, with *p* < 0.01 (df = 4, T_crit_ = 2.776, and T_stat_ = 4.816). However, there were no observed significant differences between the efficacies of D5 and D4 in CHME3 cells at 24 h of treatment (Table 3). The efficacies of D5 and D4 on the gene expression of the IKK-β enzyme, TNF-γ pro-inflammatory cytokine, and PPAR-γ anti-inflammatory cytokine demonstrated that D5 significantly reduces the expression level of IKK-β in SVG cells by around 1.702 ± 0.135, with *p* < 0.05 (df = 2, T_crit_ = 4.303, and T_stat_ = 4.48); however, there was no significant difference observed among the efficacies of D5 and D4 in CHME3 cells after 24 h of treatments (Table 3).

The same pattern was observed in the level of *TNF-α* gene expression in SVG and CHME3 influenced by 24 h treatment by D4 and D5. D5 showed a significant reduction in the level of *TNF-α* gene expression in SVG by around 2.007 ± 0.328 compared with D4, with *p* < 0.05 (df = 2, T_crit_ = 4.303, and T_stat_ = 5.684); moreover, no significant difference was found among the groups of CHME3 cells treated with D5 and D4 (Table 3). In contrast, the efficacies of D5 and D4 on the level of *PPAR-γ* gene expression showed no significant difference in the LPS-stimulated SVG cells, while in LPS-stimulated CHME3, D5 more remarkably up-regulated the expression level of the *PPAR-γ* gene after 24 h compared with D4, by around 2.153 ± 0.179 times, with *p* < 0.01 (df = 3, T_crit_ = 3.182, and T_stat_ = 15.143) (Table 3).

**Figure 11:**
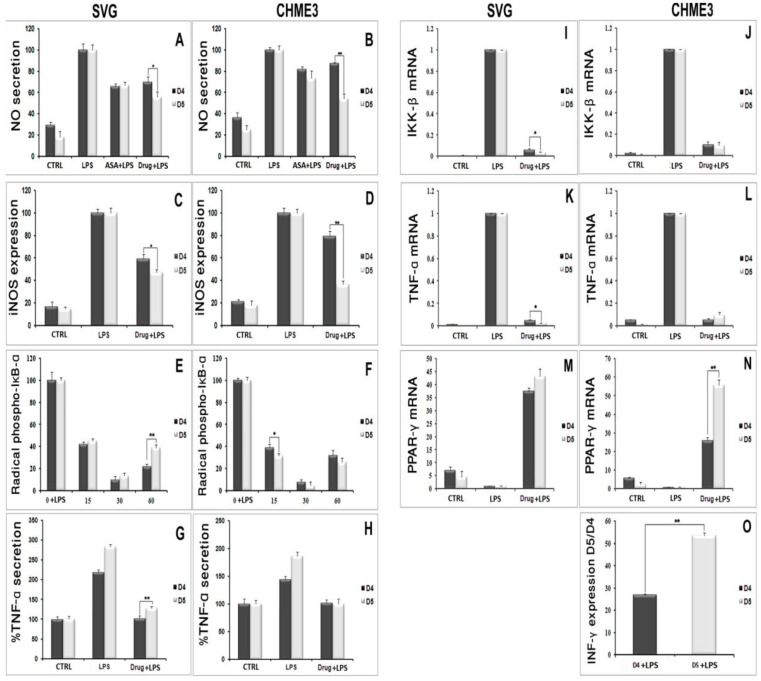
**In vitro comparison of the druggability potential of methylenedioxy and hydroxymethyl piperamide derivatives on the LPS-inducing inflammation in the astrocytic glial** (**SVG**) **and microglial** (**CHME3**) **cells**: Astrocytic glial (SVG) and microglial (CHME3) cells were treated with D4, D5, and ASA in their respective EC50 values (0.86 µM, 0.71 µM, and 52.73 µM, respectively) for 2 h, and then incubated with LPS (100 ng/mL) for 24 h. the efficacies of all compounds were compared using the pairwise Student’s *t*-test, with *p* ≤ 0.05. The in vitro tests consisted of assessing the level of NO quantification using Griess reagent ((**A**,**B**) in SVG and CHME3, respectively); protein secretion of iNOS ((**C**,**D**) in SVG and CHME3) and phospho-IκB-α ((**E**,**F**) in SVG and CHME3) using Western blot analysis;cytokine protein secretion (TNF-α ((**G**,**H**) in SVG and CHME3) and INF-γ ((**O**) in CHME3)) using Enzyme-Linked Immunosorbent Assay (ELISA); and cytokine and enzyme genes expression (IKK-β ((**I**,**J**) in SVG and CHME3), TNF-α ((**K**,**L**) in SVG and CHME3), and PPAR-γ ((**M**,**N**) in SVG and CHME3)) using quantitative real-time PCR.The Western blot and RT-PCR data were normalized by their respective level of GAPDH protein and gene expressions. The level of INF-γ in section “O” ispresented as the ratio of INF-γ secreted in drug-treated LPS-stimulated cells and their respective drug-untreated LPS-stimulated CHME3 cells. * *p* < 0.05; ** *p* < 0.01 probability.

The efficacy comparison of the drugs on inhibiting INF-γ secretion in CHME3 cells indicated that D5 more significantly reduced the secretion of INF-γ from CHME3 in the EC (0.71 µM) dosage after 24 h of treatments than D4 by around 1.901 ± 0.081, with *p* < 0.01 (df = 3, T_crit_ = 3.182, and T_stat_ = 32.443) (Table 3). The result showed no significant difference between the efficacies of D5 and D4 on IL1-β or IL-6 secretions from SVG and CHME3 at 24 h time points. The experiments in which D4 and D5 showed significant efficacy differences are presented in Figure 11. 

## 5. Discussion

The current study focused on the evaluation of the pharmacological properties of piperine-derived D5 and D4 to repress glial cells’ over-activation and the neural cytotoxicity caused by neuroinflammation. Neuroinflammation is the activation of an immune response in the CNS (brain and spinal cord) by microglia and astrocytes. Typically, neuroinflammation occurs in response to CNS injury, infection or toxins, or as a consequence of autoimmunity [55,56,57]. While transient neuroinflammatory signaling plays a protective role during development and tissue repair following injury, chronic neuroinflammation is associated with the progression of neurodegenerative diseases including Alzheimer’s disease, Parkinson’s disease, amyotrophic lateral sclerosis, and multiple sclerosis [55,56,57]. The pathological neuroinflammation associated with neurodegeneration is primarily mediated by microglia, the resident immune cells of the CNS. It is reported that LPS, by mediating Toll-like receptor 4 (TLR-4), initially activates the microglia in the central nervous system. The activated microglial cells produce proinflammatory cytokines such as TNF-α, IL-1β, prostaglandin E2 (PGE2), and NO. These cytokines are key factors in the neuroinflammatory process [55,56]. 

We observed that D5 significantly enhanced the viability of LPS-stimulated CHME3 and SVG cells. D5 treatment of LPS-stimulated glial cells remarkably alleviated the level of proinflammatory and inflammatory chemokines, cytokines’ proteins, and genes such as iNOS, nitric oxide, TNF-α protein, mRNA, IL1-β, IKK-β mRNA, phospho-IκB-α and the active NF-κB p65 subunit. In contrast, D5 treatment of LPS-stimulated glial cells could significantly increase the mRNA level of the anti-inflammatory cytokine, PPAR-γ. However, there are several weaknesses in the obtained results. Further comprehensive studies are necessary to investigate the impact of synthesized compounds on the protein, the mRNA levels of cytokines and target proteins and enzymes, as well as structural inhibitory enzymatic activities. For instance, itis necessary to investigate the inhibitory function of D4, D5, and ASA on the protein levels of PPAR-γ and IKK-β; the enzymatic activity of IKK-β; and the mRNA level of iNOS, and IL1-β.

The results of the current study are supported by previous reports on the signaling pathways of microglia and astrocyte activation [55,56,57]. It is reported that amide derivatives of piperine demonstrated significant protection against neuronal disorders in the central nervous system [58]. Reports indicate that piperamides, by blocking the monoamine oxidase (MAO), play a role in protection against Epileptogenesis and Parkinson’s disease progression [59]. Additionally, several reports showed that replacing the piperidine residue with N-aryl amines increased the efficacy of piperine on the modulation of γ-aminobutyric acid Type A (GABA A) receptors [60]. Our result correlate with the findings of previous studies. The results showed that replacing piperidine residue with N-aryl amines improves the efficacy and physicochemical properties of piperine. However, the type of N-aryl amines may result in broad spectra of efficacy and improve the physicochemical properties of a novel derivative. Therefore, in the present study, we compared the efficacy and physicochemical properties of our previously synthesized N-aryl amide derivatives of piperine, methylenedioxy(D5), and hydroxymethyl (D4) piperamides.

First, we investigated the pharmacokinetic (ADMET profile) changes caused by functional-group modification in both derivatives of piperamide (Table 2). The result showed that modifying the N-aryl piperamide functional group with methylenedioxyled to greater change in the solubility of the compound and improved absorption when it was administered orally through the buccal cavity, stomach, intestine, and bowel, compared with hydroxymethyl derivatives. The most important reason for functional-group modification is improving the absorption of a compound to increase its bioavailability. The absorption level for every orally administered drug relies on various parameters and several criteria such as Lipinski’s rule of five, Jorgensen’s Rule of Three, **QPPCaco**, **QPPMDCK**, and absorption form the buccal cavity. The distribution of a druggable compound depends on the ability of a drug to dissolve in water (aqueous solubility, **QPlogS**). A druggable molecule has to possess the capability for metabolism and undergo various metabolic reactions inside a recipient organism to be considered for the development of a novel druggable compound (**# Primary Metabolites**). Based on Jorgensen’s rule, by improving **QPPCaco**, **QPPMDCK**, and **QPlogS**, the methylenedioxy derivative showed significantly better bioavailability and absorption compared to the hydroxymethyl analog. To study aqueous solubility, various parameters have to be considered, including **SASA**, **FOSA**, **FISA**, **donorHB**, **accptHB**, and **QPlogPo/w**. Methylenedioxy, by significantly reducing the level of **SASA**, **FOSA**, **FISA**, **donorHB**, and **accptHB**, improved aqueous solubility (**QPlogS**) and, consequently, accelerated distribution through the bloodstream. The next important criterion is an excretion. If a compound is supposed to be utilized as a drug, it has to be excreted and degraded inside the body to avoid accumulation and toxicity in living organisms. The most important parameter for the estimation of excretion is the biodegradability level. The results herein indicated that methylenedioxy modification resulted in greater improvement of the bioavailability of piperamide than the hydroxymethyl functional group. The final and most critical criterion is the toxicity profile of a compound for humans and rodents. Various parameters are considered to determine the safety and druggability of a compound, including the potential toxicity of a compound for the blockage of HERG K+ channels (**QPlogHERG**); mutagenicity for rodents; carcinogenicity for rodents; skin and ocular irritancy and sensitizability; the potential to develop toxicity (DTP); the concentration of a substance that causes adverse effects on 50% (**EC50**) of the test population of *Daphnia magna*, within a selected period; carcinogenicity for humans (weight of evidence (**WOE**)); lethal dose for rodents (**LD50**); lethal concentration for rodents (**LC50**); and tolerate dose for rodents (**TD50**). Methylenedioxy functional-group modification reduced the level of toxicity for HERG K+ channels and increased the tolerance in rodents to a lethal concentration of piperamide compared to hydroxymethyl. 

This computational study of pharmacodynamic changes related to functional-group modification demonstrated that methylenedioxy did not show any improvement in the tendency of the compound for the active site of the IKK-β enzyme compared to the hydroxymethyl group. In addition, the replacement of hydroxymethyl with methylenedioxy may decrease the stability of the compound inside the active site of the enzyme. The molecular symmetry in (2E,4E)-N,5-bis(benzo[d][1,3]dioxol-5-yl)penta-2,4-dienamide (D5) may reduce molecular flexibility, influencing the proper settlement of the compound inside the IKK-β active site, and may decrease the number of hydrogen bonds between ligand and receptor.

Finally, we checked the influence of functional-group replacement on the compound’s anti-inflammatory profile through an in vitro study. We observed that by replacing the hydroxymethyl group with the methylenedioxy group, the efficacy of the compound in alleviating the protein and mRNA levels of pro-inflammatory cytokines was significantly increased in the LPS-stimulated cells. 

## 6. Conclusions

In the present study, we investigated the immunomodulatory function of a novel piperamide derivative called D5 on LPS-induced inflammation in human microglial (CHME3) and astrocytic glial (SVG) cell lines. The results indicated that D5 significantly reduced the level of pro-inflammatory cytokines such as TNF-α, INF-γ, IL-1β, and IL-6; iNOS; and chemokines such as NO. Additionally, D5 was able to significantly up-regulate *PPAR-γ* mRNA levels in the LPS-stimulated glial cells. D5, by down-regulating the mRNA level of IKK-β, significantly inhibited NF-κB activation and nuclear translocation in SVG and CHME3 cells after 24 h of incubation. The obtained data showed significant efficacy of D5 compared to ASA on the inhibition of the NF-κB pathway that leads to neuroinflammation. In addition, the comparison of the functional-group modification of piperine demonstrated that the methylenedioxy group resulted in greater improvement of the pharmacokinetics and immunomodulatory effects of piperine than the hydroxymethyl group. The present study introduces D5 as a potential anti-neuroinflammatory agent for further in vitro, in vivo, and clinical investigations.

## Figures and Tables

**Table 1:** Dose selection based on theoretical EC50, ½ EC50, and 2x EC50.

Drug	Amounts	DOSE *	IL-6/24 h		INF-γ/24 h	
			OD	QUANTIFY (pg/mL)	OD	QUANTIFY (pg/mL)
D4	½ EC50	140	0.32	282.11 ± 5.24	0.30	219.82 ± 10.13
	EC50	280	0.22	188.07 ± 6.17	0.23	163.04 ± 4.52
	2x EC50	560	0.17	141.04 ± 8.03	0.21	146.82 ± 5.85
D5	½ EC50	120	0.28	259.08 ± 9.22	0.31	291.37 ± 5.93
	EC50	240	0.21	194.93 ± 5.92	0.20	186.97 ± 5.28
	2x EC50	480	0.19	170.98 ± 5.40	0.16	149.33 ± 7.12
ASP	½ EC50	4600	0.31	283.64 ± 5.13	0.33	309.09 ± 5.23
	EC50	9100	0.27	244.04 ± 10.86	0.20	192.98 ± 6.18
	2x EC50	18,200	0.22	206.90 ± 8.05	0.14	131.92 ± 7.77
CELL + LPS			0.44	402.44 ± 8.29	0.43	412.54 ± 4.49
CELL			0.16	144.28 ± 5.99	0.12	111.68 ± 7.90

* Based on ng/mL LPS concentration (100 ng/mL).

**Table 2:** The computed physicochemical properties of D5 using the Qikprop approach of Schrodinger suite 2018.

Physicochemical Properties	Computed Range		Acceptable Range
	D5	D4	
**SASA**	565.2	611.314	300–1000
**FOSA**	196.15	179.518	0–50
**FISA**	52.860	101.941	7–330
**Volume**	993.086	1044.573	500–2000
**donorHB**	1	2	0–6
**accptHB**	5.5	5.7	2–20
**CNS**	0	−1	−2–2
**QPlogPC16**	10.272	11.365	4–18
**QPlogPoct‡**	14.632	17.079	8–35
**QPlogPw**	9.148	10.906	4–45
**QPlogPo/w**	3.232	3	−2–6.5
**QPlogHERG**	−5.44	−6.108	<−5
**QPlogBB**	−0.284	−0.943	−3–1.2
**QPPMDCK**	1694.260	531.993	<25 poor, >500 great
**QPlogKp**	−0.804	−1.468	−8–−1
**QPlogKhsa**	0.012	−0.012	−1.5–1.5
**HOA**	3	3	1–3
**%HOA**	100	100	<25% poor, >80% high
**ROF**	0	0	Max 5
**ROT**	0	0	Max 3
**QPlogS**	−3.642	−4.03	>−5.7 *
**QPPCaco**	3210.48	1069.535	>22 nm/s *
**# Primary Metabolites**	0	1	<7 *
**WOE**	Non-carcinogen	Non-carcinogen	
**Rodent Carcinogenicity**	Non-carcinogen	Non-carcinogen	
**Rodent Mutagenicity**	Non-mutagen	Non-mutagen	
**LD50**	2.152542	4.905009	g/kg_body_weight
**LC50 (inhalation)**	8.922757	8.173971	mg/m^3^/h
**TD50**	0.167370	0.196023	g/kg_body_weight
**EC50**	0.241032	0.560252	mg/L

* Jorgensen’s rule of three consists of QPlogS, QPPCaco, and # primary metabolites, known as the bioavailability likeness of an orally administered drug molecule. **SASA**: total solvent-accessible surface area (SASA) in square angstroms using a probe with a 1.4 Å radius; **FOSA**: hydrophobic component of the SASA; **FISA**: hydrophilic component of the SASA; **Volume**: total solvent-accessible volume in cubic angstroms using a probe with a 1.4 Å radius; **donorHB**: estimated number of hydrogen bonds that would be donated by the solute to water molecules in an aqueous solution; **accptHB**: estimated number of hydrogen bonds that would be accepted by the solute from water molecules in an aqueous solution; **CNS**: predicted central nervous system activity on a –2 (inactive) to +2 (active) scale; **QPlogPC16**: predicted hexadecane/gas partition coefficient; **QPlogPoct‡**: predicted octanol/gas partition coefficient; **QPlogPw**: predicted water/gas partition coefficient; **QPlogPo/w**: predicted octanol/water partition coefficient; **QPlogHERG**: predicted IC50 value for blockage of HERG K+ channels; **QPlogBB**: predicted brain/blood partition coefficient; **QPPMDCK**: predicted apparent MDCK cell permeability in nm/sec; **QPlogKp**: predicted skin permeability, log Kp; **QPlogKhsa**: prediction of binding to human serum albumin; **HOA**: predicted qualitative human oral absorption (1, 2, or 3 for low, medium, or high); **%HOA**: predicted human oral absorption on a scale of 0 to 100%; **ROF**: number of violations of Lipinski’s rule of five; **ROT**: number of violations of Jorgensen’s rule of three; **QPlogS**: predicted aqueous solubility, log S; **QPPCaco**: predicted apparent Caco-2 cell permeability in nm/sec; **# Primary Metabolites**: number of likely metabolic reactions. **WOE**: weight of evidence (carcinogenicity for humans), rodent carcinogenicity, and mutagenicity; **LD50**: lethal dose for rodents; **LC50**: lethal concentration for rodents; **TD50**: tolerated dose for rodents; and **EC50**: a concentration of a substance that causes adverse effects on 50% (EC50) of the test population of *Daphnia magna*, within a selected period, is used for predicting an optimal effective dosage of a druggable compound with minimal cytotoxic effect.

**Table 3:** Efficacy comparison between methylenedioxy (D5) and hydroxymethyl (D4) piperamides on the neuroinflammatory cytokine protein and gene.

Name	D4		D5		P_value_		T_crit_		T_stat_		df	
	SVG	CHME3	SVG	CHME3	SVG	CHME3	SVG	CHME3	SVG	CHME3	SVG	CHME3
* No secretion (normalized by LPS group)	69.9 ± 4.7	87.4 ± 1.0	55.6 ± 5.0	54.3 ± 4.4	0.05	0.01	2.8	4.3	3.6	12.7	4	2
**% iNOS secretion** (normalized by LPS group)	59.2 ± 3.9	79.2 ± 4.8	47.2 ± 2.6	36.3 ± 3.2	0.05	0.01	3.2	2.8	4.5	12.9	3	4
**% Phospho-IkB-α** (normalized by LPS group)	22.0 ± 1.8	32.1 ± 4.7	38.8 ± 2.8	25.8 ± 3.8	0.01	0.05	3.2	2.8	−8.8	3.4	3	4
**% TNF-α secretion** (normalized by LPS group)	127.7 ± 5.4	100 ± 9.2	101.7 ± 7.7	101.6 ± 6.0	0.01	--	2.8	--	4.8	--	4	--
**^#^ INF-γ secretion** (normalized by LPS group)	73.2 ± 0.5	--	46.7 ± 1.3	--	0.01	--	3.2	--	32.4	--	3	--
**^@^ IKK-β gene expression** (normalized by LPS group)	0.062 ± 0.010	0.105 ± 0.024	0.036 ± 0.003	0.098 ± 0.028	0.05	--	4.3	--	4.5	--	2	--
**^@^ TNF-α gene expression** (normalized by LPS group)	0.05 ± 0.008	0.054 ± 0.012	0.025 ± 0.001	0.096 ± 0.027	0.05	--	4.3	--	5.7	--	2	--
**^@^ PPAR-γ gene expression** (normalized by LPS group)	37.365 ± 1.347	25.909 ± 1.792	43.133 ± 2.836	55.624 ± 2.88	--	0.01	--	3.2	--	15.1	--	3

* Nitric oxide secretion (µM); ^#^ INF-γ secretion (pg/mL); ^@^ number of fold changes normalized by GAPDH and LPS group.

## Data Availability

Not applicable.
